# Mapping Nanoscale Order–Disorder Transitions to Optimal Topochemical Polymerization Across Alkyl Diacetylene Monolayers

**DOI:** 10.1002/smtd.70698

**Published:** 2026-05-07

**Authors:** Joseph A. Garfield, Soumya Paul, Shelley A. Claridge

**Affiliations:** ^1^ Department of Chemistry Purdue University West Lafayette Indiana USA; ^2^ Weldon School of Biomedical Engineering Purdue University West Lafayette Indiana USA

**Keywords:** 2‐dimensional materials, order‐disorder transitions, polydiacetylene monolayers, scanning probe microscopy, topochemical polymerization

## Abstract

On‐surface reactions within self‐assembled molecular networks offer a powerful strategy for nanoscale interface design, but must balance molecular layer stability with the local dynamics required for bond formation. Here, we develop a framework for identifying optimal reaction conditions in the topochemical polymerization of diacetylene monolayers on 2D materials. By systematically varying H‐bonding headgroup chemistry (COOH, OH, and NH_2_), we examine how bulk and surface‐confined structural transitions can be used to establish a window of optimal on‐surface polymerization efficiency. Combining temperature‐dependent polymerization measurements with atomic force microscopy, differential scanning calorimetry, and molecular dynamics simulations, we relate bulk melting measurements (*T_m_* (TCD‐COOH) = 56°C, *T_m_* (TCD‐OH) = 51°C, *T_m_* (TCD‐NH_2_) = 26°C), to observed surface‐confined structural transitions. Across all monomers, polymerization efficiency increases above room temperature with a maximum near the onset of surface disorder or near solid–solid transitions that alter distances between bond‐forming carbons (e.g., TCD‐OH herringbone phases): *T_max_* (TCD‐COOH) = 65°C, *T_max_* (TCD‐OH) = 45°C, and *T_ma_*_x_(TCD‐NH_2_) = 45°C. These results demonstrate the relationship between optimal on‐surface reactivity and pre‐melting increases in alkyl chain mobility, establishing a broader strategy for maximizing on‐surface topochemical polymerization efficiency.

## Graphical abstract

On‐surface reactions within self‐assembled molecular networks offer a powerful strategy for nanoscale interface design, but must balance molecular layer stability with the local dynamics required for bond formation. Here, we develop a framework for identifying optimal reaction conditions in topochemical polymerization of diacetylene monolayers on 2D materials, by systematically varying H‐bonding headgroup chemistry (COOH, OH, and NH_2_).

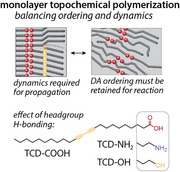

## 1 Introduction

On‐surface reactions in self‐assembled molecular networks represent a powerful strategy for customized interface design [1, 2]. However, such reactions must balance the competing requirements for molecular layer stability with local molecular dynamics necessary for bond formation. This balance is particularly critical for topochemical reactions [3], where precise molecular registry must be preserved while still allowing sufficient atomic‐scale motion to enable reaction. Diacetylene polymerization [3, 4, 5, 6, 7] represents a key class of on‐surface reactions for modular interface design [8, 9, 10, 11, 12, 13]. Diacetylene (DA) monomers are exceptionally useful, with a wide range of chemical functionalities that can be incorporated [14], spatial control and solvent‐free reaction conditions afforded by topochemical photopolymerization, and modulation of polymerization through monomer design, including alkyl chain structure [15, 16, 17, 18].

**FIGURE 1:**
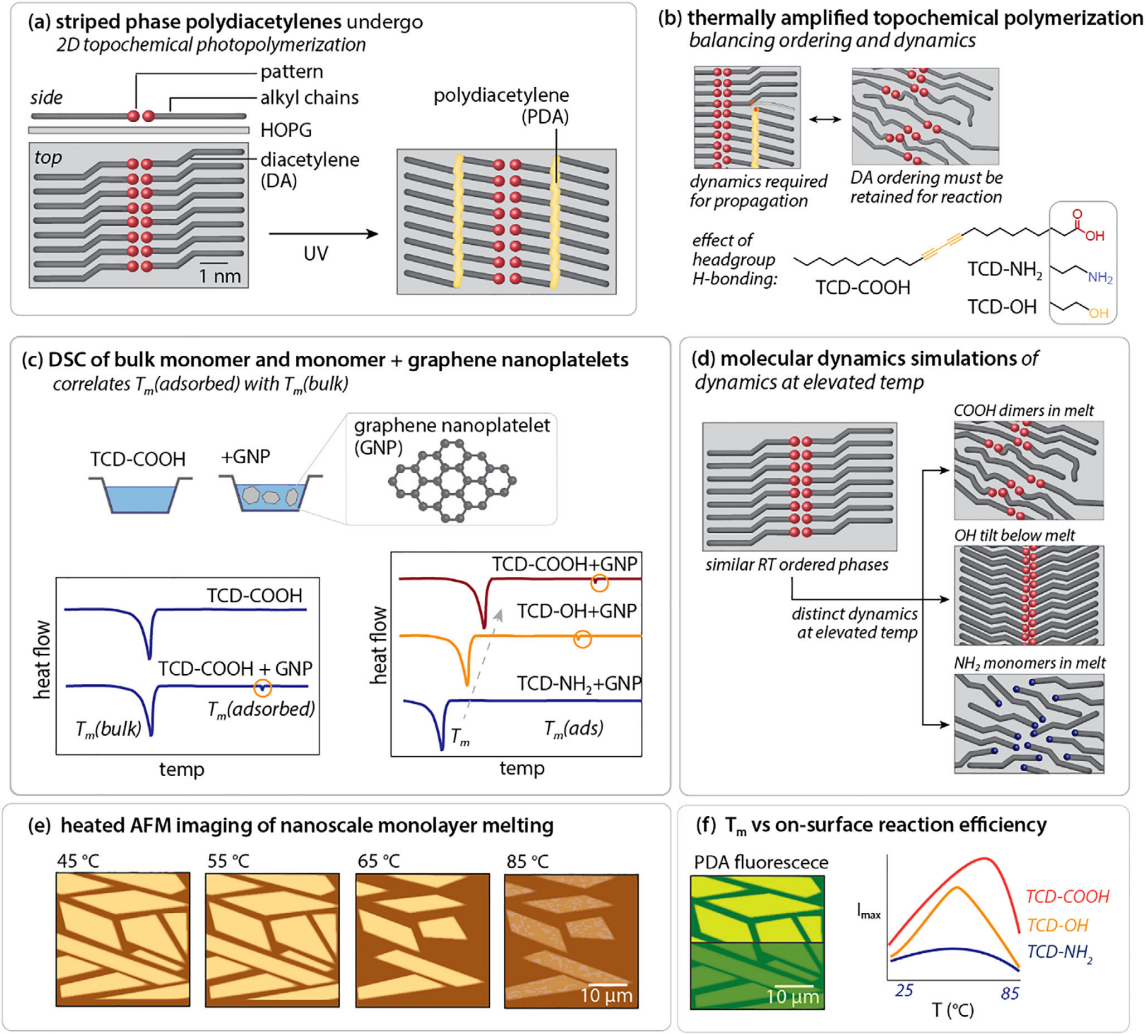
Schematic of (a) striped‐phase monolayer structure on HOPG, and (b) molecular dynamics required for topochemical polymerization, illustrating monomers chosen to compare impacts of hydrogen bonding. (c) DSC characterization of bulk and adsorbed melting transitions. (d) Molecular dynamics simulations of striped phase monolayers at elevated temperatures. (e) Heated AFM images of monolayer disordering near domain edges. (f) Functional assay of on‐surface topochemical reaction efficiency.

Although conventionally studied in 3D crystals, DA amphiphiles can also be used to generate covalently linked, nanoscale functional patterns with well‐defined geometries [19, 20]. DA monomers with long alkyl chains can assemble on highly oriented pyrolytic graphite (HOPG) with alkyl chains oriented parallel to the substrate, forming 1‐nm‐resolution functional patterns with a sub‐10‐nm pitch (Figure 1a) [8]. In this configuration, the substrate plays an important role in modulating molecular packing, dynamics, and reactivity (Figure 1b) [10, 21, 22, 23, 24, 25]. For instance, the evolution of ∼0.1‐Å high linear topographical features during polymerization of certain DA monomers is associated with the formation of a ‘lifted’ PDA backbone to minimize alkyl chain steric clashes during polymerization [8, 16]. Ordered alkyl chain segments visible in STM images after polymerization suggest initial formation of a ‘blue’ PDA backbone, while extended UV irradiation can produce monolayer structural changes interpreted as conversion to ‘red’ phase [26]. It is important to note that conventional chromic characterization is not feasible in monolayers of this type, due to very low Beer's‐law absorbance in a 0.5‐nm thick film (*calc*. 0.001–0.002 based on values for thicker multilayer films [27], see Supporting Information for more details).

In the early stages of polymerization, polymer lengths can be imaged directly for certain monomers, based on the evolution of raised PDA backbones as described above. Although polymerization efficiencies in such surface‐confined systems are typically lower than those achieved in 3D crystals, average degrees of polymerization (DP) for functional diynoic acids routinely reach 100–200 or higher for polymerization at room temperature [16, 28, 29], corresponding to polymers 50‒100 nm in length. Monomers can be patterned at the microscale using microcontact [30] or inkjet printing [31], as well as through nanoscale methods including nanoshaving [32]. Following polymerization, the covalently linked functional patterns can be transferred to a range of other materials, including polydimethylsiloxane (PDMS) [33, 34, 35, 36] and hydrogels (e.g., polyacrylamide) [37, 38], generating soft material interfaces that display nanostructured functional patterns including arrays of carbohydrates [39].

Recently, we have shown that reaction efficiency (quantified by average DP) can be substantially increased for 10,12‐tricosadiynoic acid (TCD‐COOH), by carrying out polymerization just below the onset of monolayer disorder (Figure 1b) [40]. This observation suggests that increased molecular dynamics prior to full disordering can significantly improve reaction efficiency, motivating a broader investigation into how temperature‐dependent structural transitions regulate on‐surface reactivity.

Understanding the extensibility of this approach requires establishing relationships between monomer structure and potential metrics for alkyl chain mobility, including bulk phase behavior, simulated or experimental evidence of surface‐confined structural transitions, and polymerization efficiency. Bulk and surface thermal phase transitions of simple alkanes assembled on graphite have been characterized previously, using both molecular‐scale and ensemble techniques [41, 42, 43, 44, 45, 46, 47, 48, 49, 50]. Differential scanning calorimetry (DSC) measurements of simple alkanes confined in expanded graphite indicate that the layer of alkanes nearest the graphitic surface has a T_m_ up to 50°C higher than the bulk T_m_ [44, 47].

Molecular‐scale measurements provide additional insight of potential importance for assessing the ordering of DA groups as alkyl chains begin to undergo dynamics. Scanning tunneling microscopy (STM) studies illustrate that, for simple straight‐chain alkanes, melting proceeds through lateral roughening of the lamellae, with molecules becoming offset relative to the lamellar axis [43]. For diacetylene (DA) monomers, the shape complementary enforced by the DA unit would limit edge roughening, meaning that thermal transitions observed for simple alkanes do not directly translate to DA alkanes of similar molecular weight. In addition, STM studies of alkyl monomers with differing functional headgroups have illustrated differences in packing arrangement (e.g., tilted or herringbone phases) [21, 51, 52] that also modulate monolayer behavior. Scanning probe measurements have also shown that alkyl chain structure plays multiple roles in DA polymerization, contributing to ordering, as well as inducing Angstrom‐scale lifting of the PDA backbone in the molecular layer contacting the substrate [8, 26, 28, 53]. While phase transitions of DA monomers have been studied in bulk by DSC [13, 54, 55, 56, 57], there is limited insight into temperature‐dependent behavior on HOPG.

Here, we examine the role of headgroup interactions in regulating polymerization at elevated temperatures, using structurally analogous diacetylene monomers 10,12‐tricosadiynamine (TCD‐NH_2_) and 10,12‐tricosadiyn‐1‐ol (TCD‐OH), benchmarked against TCD‐COOH. Differences in hydrogen‐bonding strength between these headgroups (2.3 kcal/mol for NH_2_ pairing [58, 59] and 4.3 kcal/mol for OH pairs [60], vs. 7−8 kcal/mol for COOH dimers [61]) are expected to systematically alter striped phase stability and temperatures of surface structural transitions, which we benchmark using DSC measurement of both pure monomer and monomer in the presence of graphene nanoplatelets (Figure 1c). Striped phases of hydroxyl alkanes can also form tilted or herringbone packing arrangements that optimize lateral (catenated) H‐bonding, at the expense of diacetylene packing and reactivity [51].

Using this family of monomers enables us to examine multiple distinct mechanisms through which headgroup chemistry modulates monolayer dynamics governing photopolymerization at elevated temperatures (Figure 1d). Models are correlated with nanoscale observations of monolayer disordering in elevated‐temperature AFM measurements (Figure 1e), and microscale metrics of on‐surface reaction efficiency (Figure 1f), providing an integrated framework for understanding impacts of temperature on surface reaction efficiency.

## 2 Results and Discussion

### 2.1 Simulations of Monolayer Disorder Transitions

To estimate surface order‒disorder transition temperatures for monomers used in subsequent experiments, molecular dynamics (MD) simulations were performed from 25°C–95°C. Because simulations of defect‐free crystals typically overestimate disorder transition temperatures [62, 63], we adapted a method developed previously by others to study melting in atomic and small‐molecule crystals [64], in which voids are introduced into the crystal to nucleate the phase transition (Figures S1‒S4).

**FIGURE 2:**
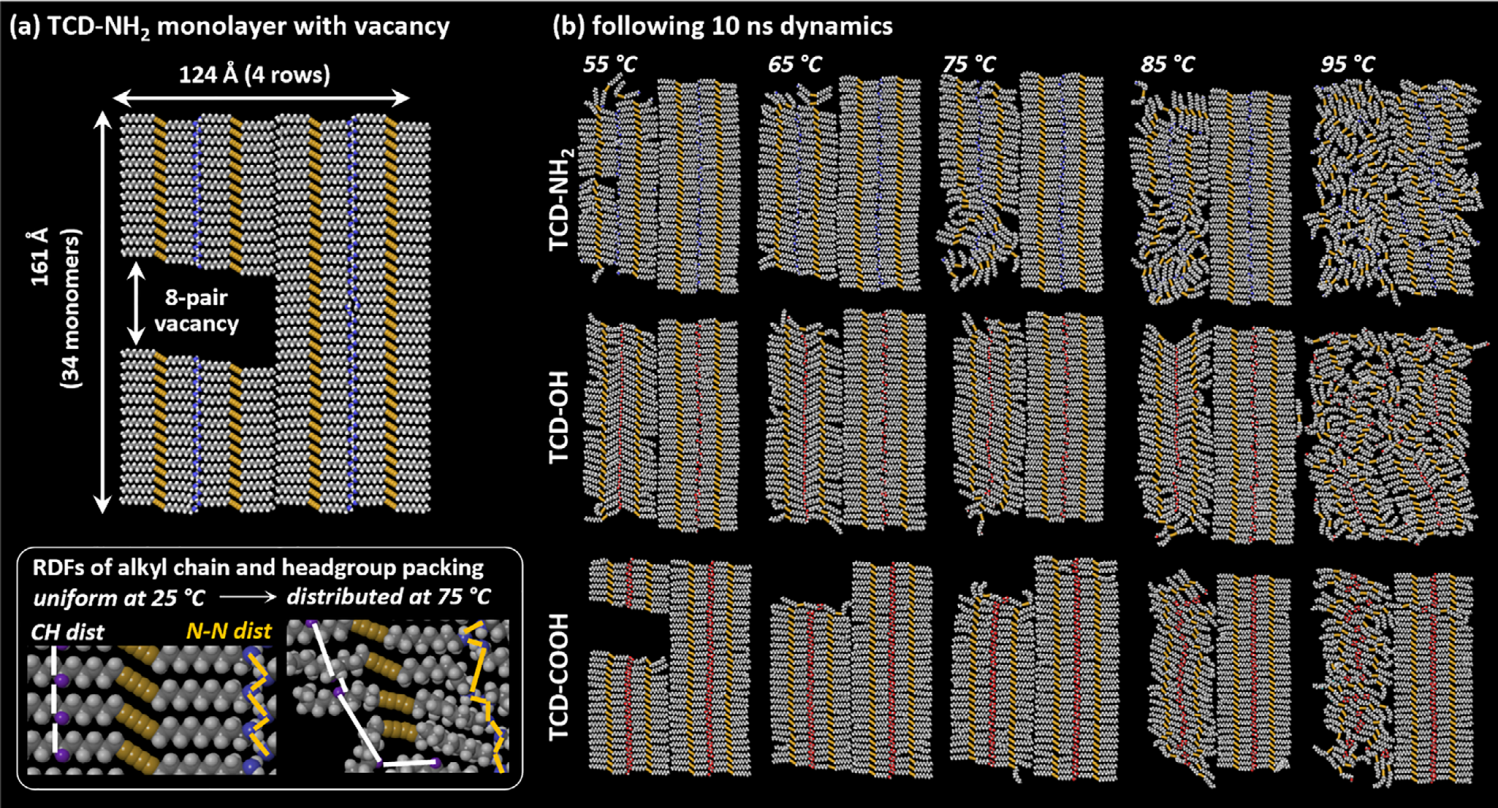
(a) Model of a TCD‐NH_2_ monolayer containing an eight‐pair vacancy, illustrating the initial structure of the tightly packed and vacancy‐containing rows used as input for molecular dynamics simulations. (b) Representative frames from 10 ns molecular dynamics simulations of TCD‐NH_2_ (top row), TCD‐OH (middle row), and TCD‐COOH (bottom row) at the temperature indicated at the top of each column (55°C–95°C). In all cases, the two vacancy‐containing rows are shown on the left, adjacent to two tightly packed rows on the right. Note that, during simulations, vacancies may migrate, reorganize, or be eliminated due to dynamics. Inset (lower left) illustrates atomic selections used to compute radial distribution functions in Figure 3. Hydrogens on the C20 carbon of each alkyl chain oriented away from the HOPG surface (purple in inset) were used to quantify alkyl chain dynamics (C–H RDFs), while headgroup heteroatoms (N shown in blue) were used to assess headgroup ordering. Gold lines highlight changes in nearest‐neighbor distances between the initial ordered configuration (left) and a representative structure after 10 ns of dynamics at 75°C (right).

A universal monolayer cell consisting of four molecular rows on HOPG was constructed, with 34 monomers per row. Eight pairs of monomers were removed from the two left rows to create vacancies (Figure 2a). Periodic boundary conditions were applied in both lateral directions, allowing molecular migration across boundaries and producing configurations such as those shown in Figure 2b.

Snapshots after 10 ns reveal pronounced differences in thermal stability as temperature increases from 55°C to 95°C (Figure 2b). For TCD‐NH_2_ (top row) molecules adjacent to vacancies become disordered at relatively low temperatures, with substantial loss of row registry by 65°C–75°C and extensive disruption of both vacancy and tightly packed rows at 95°C. TCD‐OH monolayers (center row) retain order in vacancy rows up to 85°C, although tilting begins near 55°C, producing a herringbone arrangement that optimizes catenated H‐bonding along the row. At 95°C, both vacancy and tightly packed rows become largely disordered.

TCD‐COOH (bottom) exhibits the highest thermal stability. Molecular ordering is largely preserved up to 85°C, with vacancy rows showing increased tilting and partial disorder at this temperature. Clear disorder emerges at 95°C in vacancy rows, while tightly packed rows remain mostly ordered on the 10 ns timescale, consistent with stabilization by COOH dimers.

**FIGURE 3:**
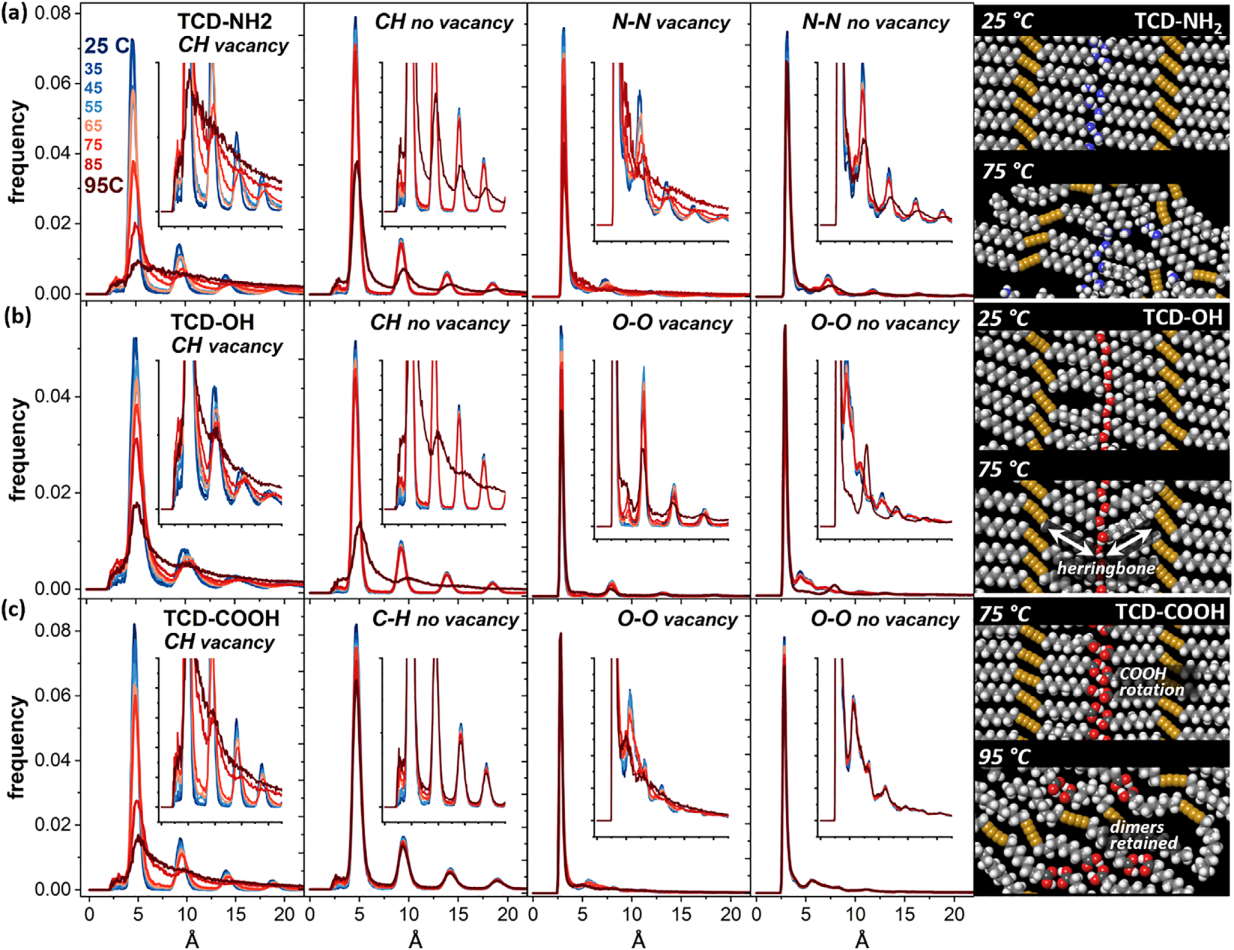
Radial distribution functions (RDFs) and representative molecular models illustrating temperature‐dependent dynamics of (a) TCD‐NH_2_, (b) TCD‐OH, and (c) TCD‐COOH monolayers. Insets within each RDF expand the low intensity region near the baseline to highlight changes in peak structure. The left two columns of RDFs quantify alkyl chain dynamics using the C20 hydrogens identified in the inset of Figure 2. The first column corresponds to vacancy‐containing rows; the second column corresponds to tightly packed rows. The third and fourth columns show headgroup dynamics, using heteroatoms (N or O) as indicated in Figure 2, for vacancy‐containing rows (third column) and tightly packed rows (fourth column). Molecular models at the far right illustrate differences in packing within vacancy‐containing rows after 10 ns of dynamics, shown at 25°C (top of each panel) and at 75°C or 95°C (bottom of each panel).

To separate the roles of alkyl‐chain packing and headgroup interactions, radial distribution functions (RDFs) were extracted from frames from 5–10 ns (Figure 3). For each monomer, RDFs capture alkyl chain correlations in vacancy and tightly packed rows, as well as headgroup–headgroup correlations in both environments. Colored traces indicate increasing temperature from 25°C (dark blue) to 95°C (dark red). Insets expand the vertical scale near the baseline to highlight changes beyond the prominent nearest‐neighbor (nn) peak at ∼5 Å.

For TCD‐NH_2_ (Figure 3a), alkyl chains in vacancy rows begin to disorder at ∼55°C, evidenced by decreased nn peak intensity, peak broadening, and the emergence of a shorter‐distance shoulder characteristic of chain rotation. In contrast, chains in tightly packed rows remain ordered up to 95°C. N–N RDFs show analogous temperature‐dependent broadening, and snapshots indicate that disordering events frequently involve disruption of relatively weak N─N H‐bonds.

For TCD‐OH (Figure 3b), alkyl chain ordering also decreases with temperature, but appreciable peak structure persists even at 95°C in vacancy rows, while tightly packed rows remain ordered. O─O correlations are more evident in vacancy rows, consistent with catenated along‐row H‐bonding optimized in tilted or herringbone geometries adopted at lower packing densities.

TCD‐COOH (Figure 3c) exhibits alkyl‐chain disordering in vacancy rows at elevated temperatures, but tightly packed rows remain highly ordered even at 95°C. O─O correlations are broader than for TCD‐OH, reflecting rotational motion of COOH dimers rather than dissociation; notably, dimers are retained during disordering events, consistent with reduced surface mobility and enhanced thermal stability.

### 2.2 Melting Behavior of Bulk vs. Adsorbed Phase

Strong interfacial interactions between *n*‐alkanes and the graphite basal plane are known to elevate the melting temperature of surface‐adsorbed layers (T_m,surf_) relative to the bulk melting temperature (T_*m,bulk*_) [44]. To probe the thermodynamic behavior of adsorbed diacetylene amphiphiles, we performed DSC measurements on samples with and without graphene nanoplatelets (+GNP and –GNP, respectively) (Figure 4; Figures S5–S14).

**FIGURE 4:**
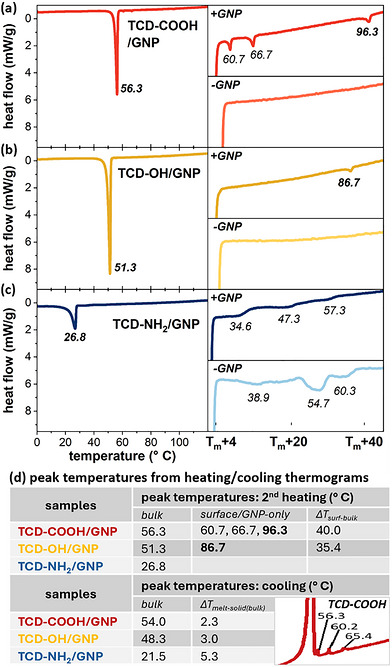
DSC thermograms from the second heating cycle for (a) TCD‐COOH, (b) TCD‐OH, and (c) TCD‐NH_2_. Left panels show full DSC traces for samples containing graphene nanoplatelets (+GNP). Right panels show expanded views of the baseline region immediately above the bulk melting transition, comparing samples with graphene nanoplatelets (+GNP, top right) and without graphene nanoplatelets (–GNP, bottom right), highlighting surface‐associated thermal transitions present only in the +GNP samples. (d) Comparison of peak temperatures from heating and cooling thermograms.

For +GNP samples, approximately 5 mg of graphene nanoplatelets were combined with an amount of amphiphile corresponding to ∼10 molecular layers, estimated using the manufacturer's reported surface area per gram (90‒130 m^2^ g^−1^). Use of high surface area GNPs ensured sufficient surface area to detect adsorbed phase transitions. DSC measurements employed an initial heating and slow cooling cycle to minimize the influence of metastable ordering, followed by a second heating cycle analysis (see Supporting Information for details). Data shown in Figure 4 correspond to the second heating cycle.

The DSC thermogram for TCD‐COOH+GNP (Figure 4a) exhibits a dominant endothermic peak at 56.3°C, corresponding to T_*m,bulk*_. In addition, several smaller endothermic features appear at 60.7°C, 66.7°C, and 96.3°C (Figure 4a, top right), which are absent in the –GNP control (Figure 4a, bottom right). These higher‐temperature transitions fall within the range previously associated with the melting of alkane layers adsorbed on graphite [44, 47]. The highest‐temperature peak at 96.3°C is consistent with melting of the layer directly in contact with the graphitic surface, yielding ΔT_*m,surf‐bulk*_(TCD‐COOH) = 40°C. Earlier work by Espeau on straight‐chain alkanes [44] identified a shoulder on the upper edge of the main melting peak that was attributed to the melting of near‐surface layers; the small peaks from 60‒70°C may represent similar transitions, or may represent transitions related to other interactions of the COOH amphiphile with the GNP surface.

For TCD‐OH+GNP (Figure 4b), both the bulk and surface melting temperatures are shifted to lower values, with T*_m,bulk_* = 51.3°C and T*_m,surf_* = 86.7°C. This reduction in comparison with TCD‐COOH is consistent with weaker hydrogen bonding between OH headgroups relative to COOH acids. Notably, the +GNP sample exhibits a single surface‐associated endotherm distinct from the –GNP bulk transition, giving ΔT*_m,surf‐bul__k_* (TCD‐OH) = 35.4°C, slightly smaller than that observed for TCD‐COOH.

TCD‐NH_2_ displays qualitatively different behavior (Figure 4c). The bulk melting temperature is substantially lower (T*_m,bulk_* = 27.9°C), and the associated endotherm is much weaker (∼2 mW g^−^^1^) than those observed for the OH and COOH analogues (∼8 mW g^−^^1^). Both +GNP and –GNP samples show a series of small endothermic features spanning ∼10°C–30°C above T*_m,bulk_* with significantly decreased peak magnitudes in the +GNP sample. We attribute these features to minor impurities or partial amine ionization that alters headgroup pairing and associated thermal transitions. The positioning of these peaks complicates the assignment of a distinct surface‐melting temperature for TCD‐NH_2_.

A summary of melting and solidification temperatures extracted from second‐cycle heating and cooling thermograms is shown in Figure 4d (top and bottom, respectively). For TCD‐COOH and TCD‐OH, the bulk‐to‐surface melting temperature shifts are comparable (∼40°C and ∼35°C, respectively), consistent with a strong stabilizing effect of adsorption to graphitic surfaces that restricts alkyl chain dynamics. Cooling thermograms exhibit slightly lower transition temperatures than the corresponding bulk melting peaks (2°C‒5°C). In the case of TCD‐COOH+GNP, three small exothermic features are also observed at 56.3°C, 60.2°C, and 65.4°C during cooling (Figure 4d, bottom right).

Surface‐adsorbed melting temperatures extracted from DSC are in reasonable agreement with disorder transition temperatures predicted by MD simulations. For TCD‐COOH and TCD‐OH, simulations indicate the onset of significant disorder in vacancy‐containing rows between ∼85°C and 95°C, similar to the highest‐temperature endotherms assigned to surface‐melting transitions in the +GNP samples. This correspondence supports the interpretation that the simulated disorder transition reflects a surface‐melting process rather than complete desorption or bulk melting. In contrast, the broader and less well‐defined thermal features observed for TCD‐NH_2_ are consistent with MD predictions of earlier and more heterogeneous disordering, reflecting weaker and less directional headgroup interactions that complicate assignment of a single surface melting temperature.

### 2.3 Nanoscale Measurements of Monolayer Dynamics

The adsorbed layer transitions measured by DSC correspond to ensembles of adsorbed layers and may therefore differ from disordering behavior in an isolated monolayer. To directly probe the temperature range over which monolayers lose structural order, we performed repeated AFM imaging in individual areas of samples while incrementally increasing sample temperature (see Figures S15‒S21 details).

**FIGURE 5:**
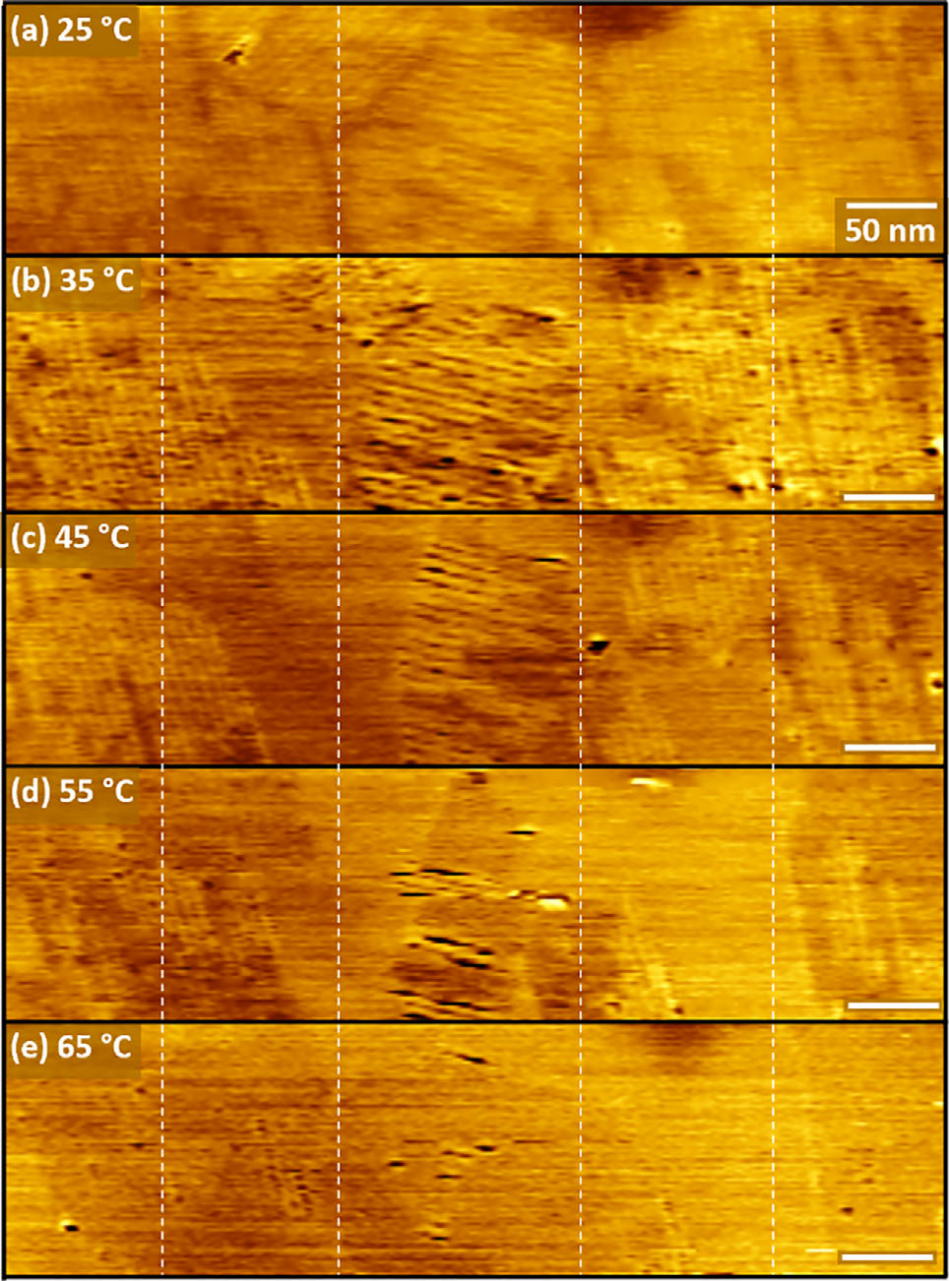
AFM height images of unpolymerized TCD‐NH_2_ on HOPG at (a) 25°C, (b) 35°C, (c) 45°C, (d) 55°C, and (e) 65°C. Vertical dashed lines are to facilitate visual comparison of regions that undergo ordering changes at higher temperatures.

Unpolymerized TCD‐NH_2_ monolayers on HOPG were imaged at setpoint temperatures from 25°C‒65°C (Figure 5a–e). At 25°C, lamellar domains are readily resolved, with high surface coverage and small vacancies localized primarily at domain boundaries (Figure 5a). In contrast to TCD‐COOH monolayers, which remain stable up to ∼75°C, TCD‐NH_2_ exhibits pronounced domain edge degradation and reduced lamellar regularity at temperatures as low as 35°C (Figure 5b), indicating an early onset of structural dynamics. Progressive surface restructuring is observed at 45°C (Figure 5c) and 55°C (Figure 5d), and by 65°C only limited residual ordering remains (Figure 5e); returning samples to room temperature does not restore molecular ordering. A similar temperature‐dependent loss of order is observed in partially polymerized TCD‐NH_2_ monolayers (Figure S16), indicating that polymerization does not suppress thermally driven disordering. The disordering range observed by AFM aligns with molecular dynamics predictions of early vacancy‐row instability for TCD‐NH_2_.

**FIGURE 6:**
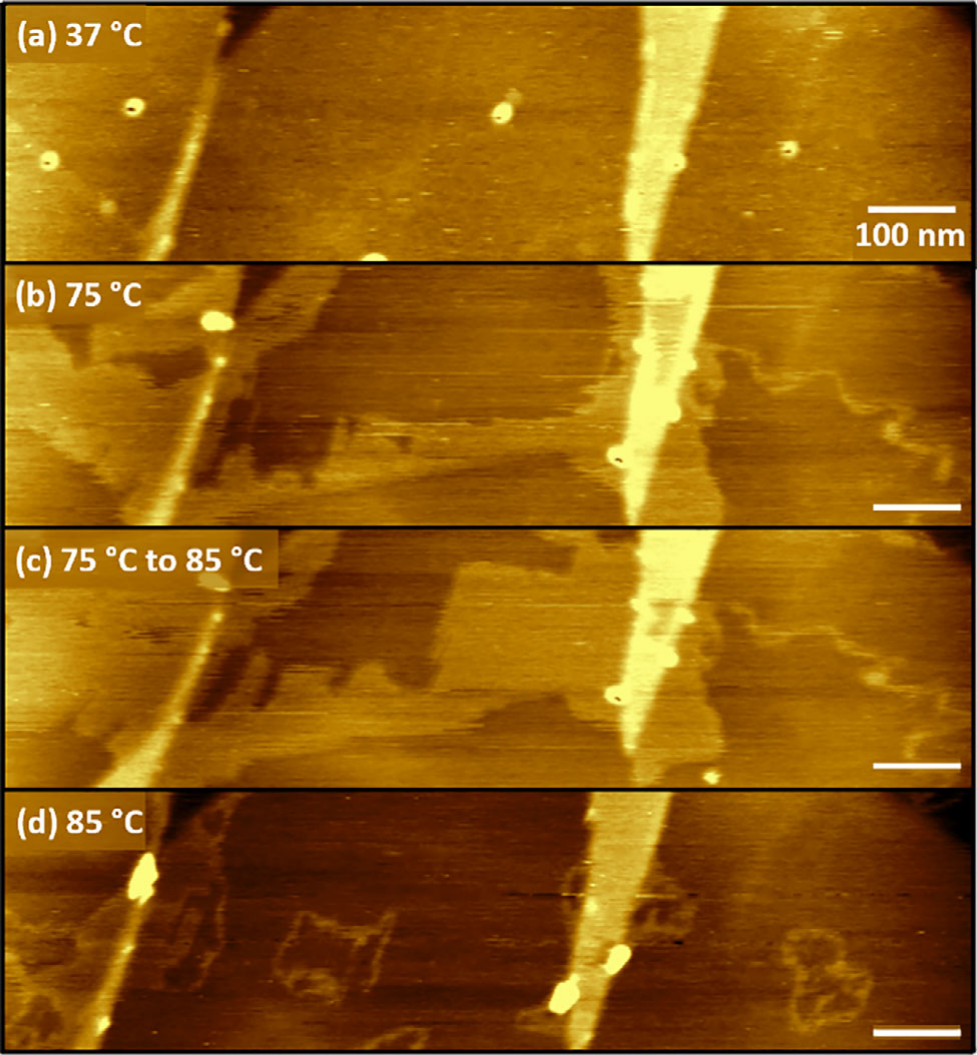
AFM height images of unpolymerized TCD‐OH on HOPG at (a) 37°C, (b) 75°C, (c) 75°C to 85°C, and (d) 85°C.

We next examined the thermal stability of TCD‐OH monolayers (Figure 6). Although lamellar ordering is not well resolved by AFM even at 37°C (Figure 6a), distinct topographic changes emerge near 75°C (Figure 6b), consistent with the onset of disordering at domain boundaries. These regions evolve rapidly as the temperature is increased from 75°C to 85°C (Figure 6c), and by 85°C most areas of the monolayer appear disordered (Figure 6d). This higher disordering temperature relative to TCD‐NH_2_ mirrors trends observed in both DSC measurements and molecular dynamics simulations, reflecting the stabilizing influence of OH headgroup interactions and the delayed onset of vacancy‐row melting.

### 2.4 Comparison of Estimates for *T_m_* and Maximum Reaction Efficiency

**FIGURE 7:**
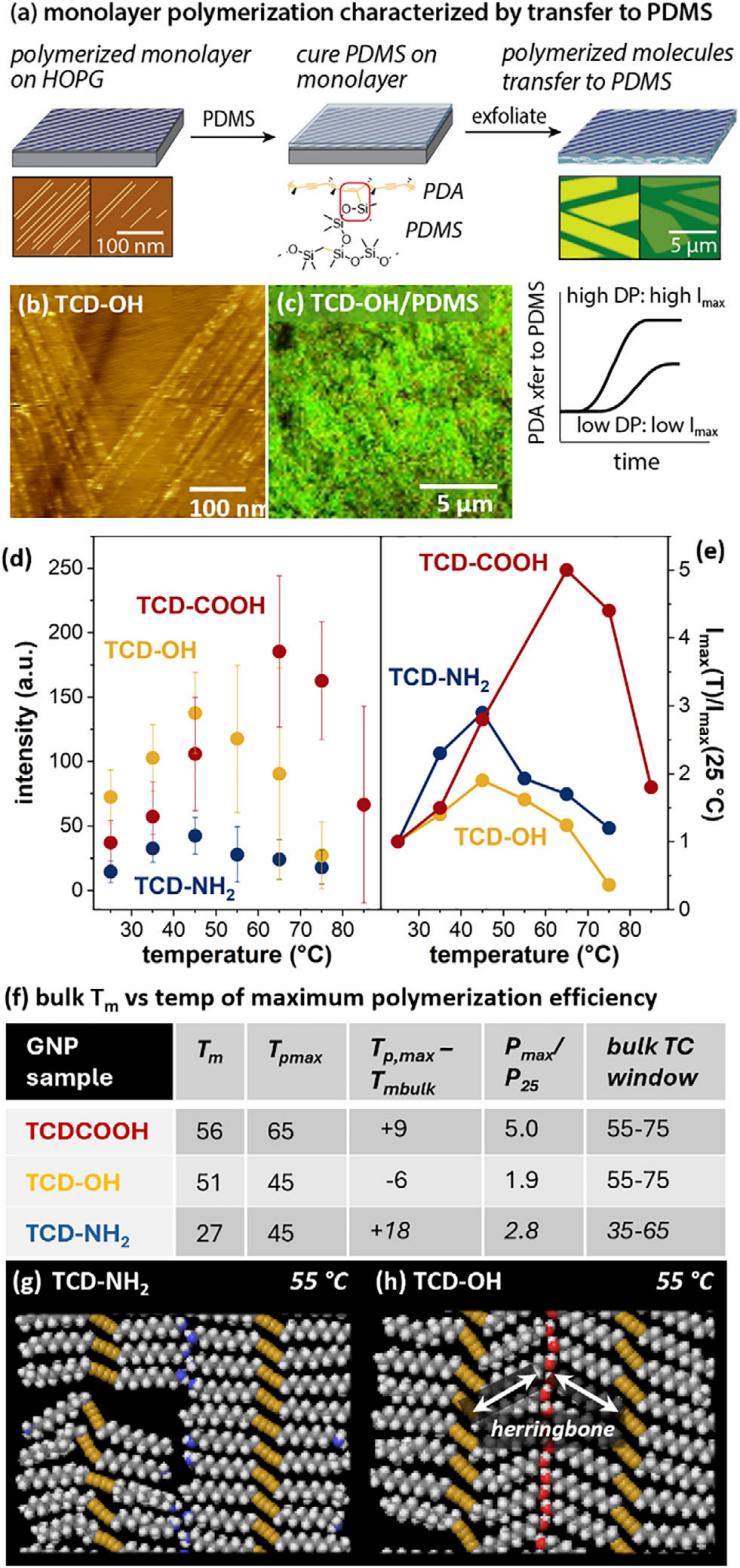
(a) Schematic illustrating the use of PDA transfer to PDMS as a comparative measurement of polymerization efficiency (taken to be DP). (b) AFM image illustrating lifted PDA backbones for partly‐polymerized monolayer of TCD‐OH on HOPG. (c) Confocal micrograph of polymerized TCD‐OH monolayer covalently transferred to PDMS, illustrating fluorescence at PDA emission maximum. (d) Plot of fluorescence peak emission of sPDA transferred to PDMS after polymerization at the indicated temperature, for TCD‐NH2, TCD‐OH, and TCD‐COOH. COOH data reproduced with permission from 34 [40]. with permission, copyright 2025 Wiley VCH. (e) Maximum fluorescence intensity normalized to the intensity value at 25°C. (f) Comparison of bulk melting temperatures, temperatures of maximum polymerization efficiency, ratios of polymerization efficiency, and bulk thermochromic window. (g) Snapshot of TCD‐NH_2_ after 10 ns dynamics at 55°C. (h) Snapshot of TCD‐OH dynamics after 10 ns at 55°C.

We next examined the relationship between T_*m,bulk*,_ and the temperature of maximum polymerization efficiency (T_p_,_max_) for each monomer (Figure 7). Polymer formation can be directly visualized in AFM images (Figure 7a, left; Figure 7b), but quantitative analysis by AFM is labor‐intensive and becomes challenging as polymer density increases, making it difficult to extract representative polymer lengths at each temperature near full conversion. To enable quantitative comparison, we instead used a previously established approach [16, 28, 65] in which monolayers polymerized on HOPG are covalently crosslinked to PDMS (Figure 7a, center) and subsequently exfoliated (Figure 7a, right). This process transfers the PDAs to PDMS, allowing fluorescence emission to be measured (Figure 7c).

Importantly, as shown in our prior work, the probability of covalent crosslinking to PDMS increases strongly with PDA length [16, 28, 65]. As a result, monolayers that form longer polymers yield higher fluorescence intensity at the reaction endpoint (I_max_) than those that form shorter polymers, even when both systems appear fully polymerized by AFM. Thus, I_max_ serves as a practical proxy for the average degree of polymerization (DP, a metric of polymer length), and therefore reflects the efficiency of the propagation step rather than the extent of monomer conversion.

Figure 7d,e compare endpoint fluorescence (I_max_) for the three monomers as a function of polymerization temperature (25–85°C); see Figures S22 and S23 for full TCD‐OH and TCD‐NH_2_ spectra. Each monomer exhibits greater polymerization efficiency at elevated temperatures. The maximum achieved polymerization efficiency increases with increasing H‐bond strength: I_max_(NH_2_) = 40 a.u., I_max_(OH) = 140 a.u., I_max_(COOH) = 190 a.u. Interestingly, however, if the data for each monomer are normalized to the efficiency of room temperature polymerization for that monomer (Figure 7e), the *proportionality* of the increase at the maximum is lower for amines (3×) than for carboxylic acids (5×), but is in fact lowest for the hydroxyl monolayers (2×). Additionally, the temperatures of maximum polymerization efficiency were the same for the hydroxyl monolayers as for the amines (T_pmax_ = 45°C for NH_2_ and OH, vs. 65°C for COOH).

Figure 7f contextualizes these observations using the bulk and surface melting data from Figures 4, 5, 6, as well as MD simulations and approximate thermochomic transition windows for each polymer in multilayer (optically thick) films (see Figure S24 for details). Carboxylic acid and amine layers exhibit differences in melting and polymerization efficiency that would largely be predicted based on differences in headgroup H‐bonding strength: the bulk melting temperature of TCD‐NH_2_ (27°C) is lower than that for TCD‐COOH (56°C), as is its T_pmax_ (45°C vs. 65°C). In each case, T_pmax_ is 10‒20°C above T_*m,bulk*_, and near the center of the multilayer thermochromic transition window (note that polymerization efficiency data and thermochromic window data are acquired at 10°C intervals).

Conversely, the measured T_pmax_ for TCD‐OH (45°C) is slightly *below* the T_*m,bulk*_ (51°C), and the multilayer thermochromic transition window, even though the monolayers retain visible ordering up to 75°C based on AFM. Notably, both prior room‐temperature STM observations and our MD simulations here indicate the existence of a herringbone polymorph for TCD‐OH, which our MD simulations suggest may become more abundant above 25°C. Thus, it would be reasonable to attribute the anomalous behavior of TCD‐OH to the formation of the less‐polymerizable herringbone polymorph.

## 3 Conclusions and Prospects

These findings demonstrate that polymerization efficiency is maximized within a temperature window defined by interfacial stabilization above the bulk melt, in the absence of competing ordered phases with less favorable DA packing for polymerization. Bulk melting temperatures provide a useful reference point for identifying the lower bound for this window, with an expected polymerization maximum 10°C‒20°C higher. However, the position of maximum polymerization efficiency and extent of thermal acceleration are also governed by nanoscale packing constraints and headgroup‐specific interactions that determine how disorder is generated at the interface.

OH‐terminated monolayers, in particular, illustrate that alternative packing can limit reactivity despite increased alkyl chain dynamics. In these monolayers, the emergence of structurally stable but less reactive arrangements, such as herringbone phases, limits increases in polymerization efficiency at elevated temperatures. By contrast, NH_2_‐terminated monolayers, characterized by substantially lower T_*m,bulk*_, also undergo more gradual disordering upon heating, leading to a progressive loss of reactive alignment and a corresponding decline in polymerization efficiency.

Taken together, these observations illustrate the value of combining straightforward ensemble DSC measurements of monomers mixed with high‐surface‐area graphene nanoplatelets (which provide lower and upper bounds for the maximum polymerization efficiency window), with MD simulations (or STM) data that provide molecular‐level insight. Heated AFM imaging, while providing usefully direct, nanoscopic insight into molecular domain disordering, is also experimentally challenging to implement with a number of samples that provide statistical insight. Overall, this framework provides a general strategy for identifying and tuning optimal reaction conditions across chemically distinct monolayer systems.

## 4 Experimental Methods

See Supporting Information for detailed Materials and Experimental Methods.

## Conflicts of Interest

The authors declare no conflicts of interest.

## Supporting information




**Supporting File**: smtd70698‐sup‐0001‐SuppMat.docx.

## Data Availability

The data that support the findings of this study are available in the supplementary material of this article.
