# Supplementary material for: Mapping Nanoscale Order–Disorder Transitions to Optimal Topochemical Polymerization Across Alkyl Diacetylene Monolayers
Source: Small Methods. 2026 May 7;10(12):e70698. doi: 10.1002/smtd.70698 (PMC13288019; doi:10.1002/smtd.70698)
Supplement: Supplementary file 1 — Supporting File: smtd70698‐sup‐0001‐SuppMat.docx. [file SMTD-10-e70698-s001.docx]

Supporting Information

**Mapping nanoscale order–disorder transitions to optimal topochemical polymerization across alkyl diacetylene monolayers**

Joseph A. Garfield†, Soumya Paul†, Shelley A. Claridge*,†,‡

†Department of Chemistry, Purdue University, West Lafayette, Indiana 47907, United States

‡Weldon School of Biomedical Engineering, Purdue University, West Lafayette, Indiana 47907, United States

*Address correspondence to: claridge@purdue.edu, (phone) 765-494-6070

**Materials.**10,12-tricosadiynoic acid (≥98 %), chloroform (contains 100-200 ppm amylenes as stabilizer, ≥99.5%), oxalyl chloride (≥99%), lithium aluminum hydride (LiAlH_4_, 95%), anhydrous dichloromethane (DCM, ≥98.8%), anhydrous diethyl ether (≥99.7%), and ammonium hydroxide (28-30%, NH_3_ basis) were all purchased from Millipore Sigma (St. Louis, MO) and used as received. Ethanol (200 proof) was purchased from Decon Laboratories Inc. (King of Prussia, PA). Anhydrous sodium sulfate (≥99%), N-N-dimethylformamide (DMF ≥99.8%), tetrahydrofuran (THF ≥99.8%), hydrochloric acid, and sodium hydroxide were purchased from Fisher Scientific (Hampton, NH). Aluminum sample pans (DSC64001) and lids (DSC64002) were purchased from DSC Consumables Incorporated (Austin, MN). Graphene Nanoplatelets (GNNP0201, 1-5 nm thickness, BET Surface Area 90-130 m^2^g^-1^) were purchased from ACS Material LLC (Pasadena, CA). Sylgard 184 (DOW manufacturer) elastomer kits, containing flowable silicone elastomer base and crosslinker, were purchased from Newark (Richfield, OH). AFM probes, Bruker RFESP-75 (0.01–0.025 Ω · cm Antimony (n)-doped Si, nominal force constant 3 N/m and radius of curvature <12 nm) were purchased from Bruker AFM Probes (Camarillo, CA). Highly oriented pyrolytic graphite (HOPG) substrates, grade ZYB, were purchased from SPI Supplies (West Chester, PA). 25-mm PTFE syringe filters were acquired from VWR (Radnor, PA) and used to filter amphiphiles. Control Cure UV filter sleeve 4’ amber for T4 fluorescent bulbs purchased from UV Process Supply Inc and used to filter laboratory lighting. Milli-Q water (≥18.2 MΩ ∙ cm resistivity) was used in all experiments where water was required. Peltier plate assembly equipment including the cold plate cooler (CP-031HT, 12 V-DC), moisture resistant thermistor with 900 mm long 26 awg wire leads (MP-3193) and thermometric temperature controller (TC-48-20) were purchased from TE Technology Incorporation (Traverse City, MI). Power supply for cold plate cooler (LRS-150F-12, AC-DC converter 1 output 12 V, 85-264 VAC, 120-370 VDC input), and toggle switch DPDT panel mount (2057-SW-T1-4B-B-K2A-ND, used to transition from hot to cold mode), were purchased from DigiKey (Thief River Falls, MN). Handheld UV lamp (UVP 95-0201-01 Model UVLS-28 EL Series 2UV, 8 Watt, 254 nm shortwave/365 nm longwave, 14.8-inch length, 115 V) was purchased from Capitol Scientific (Austin, TX). Ultraviolet photon flux was recorded using a TR-74UI-S High Precision Illuminance, UV, Temperature and Humidity Data logger. Relative humidity was measured using an Onset HOBO MX1101 Bluetooth Temperature and Humidity Data Logger. Adhesive Tape was purchased from DigiKey (P/N: 1067-9472LE-0.5-60-ND).

**Synthesis of 10,12-tricosalkyldiyn-1-ol (TCD-OH) from 10,12-tricosadiynoic acid (TCD-COOH).** Synthesis was performed based on a previously reported procedure^1^ with minor modifications described here. The starting material, TCD-COOH (1 equiv), was dissolved in anhydrous diethyl ether and placed under N_2_. The solution was then cooled to 0 °C before adding lithium aluminum hydride (3 equiv), then stirred for 4 h at room temperature. Upon completion, an ice bath was used to cool the reaction mixture to 4 °C, and the mixture was then removed from the N_2_ atmosphere. With the septum cap off, the reaction mixture was quenched with a slow addition of sodium hydroxide (15% w/w), followed by washing with water. The orange precipitate was filtered out and washed with 10% hydrochloric acid. The collected organic phase was dried with sodium sulfate (Na_2_SO_4_) and evaporated under reduced pressure to collect the desired product, TCD-OH, which appears as a white solid. See Figure S26 for ^1^H NMR spectrum.

**Synthesis of 10,12-tricosalkyldiynamine (TCD-NH_2_) from 10,12-tricosadiynoic acid (TCD-COOH).** Synthesis was carried out in accordance with previously reported literature^2-3^ with minor modifications described here. First, TCD-COOH was dissolved in anhydrous DCM and maintained under N_2_. Next, oxalyl chloride (1.3 equiv.) and a few drops of DMF were added to the solution. The reaction was stirred overnight at room temperature; the product 10,12-diynoyl chloride, a yellow oil, was recovered and used without further purification. In a round bottom flask, 28-30% ammonium hydroxide (1.3 equiv.) was added, followed by 10,12-diynoyl chloride (1 equiv.) in THF that was cooled to 0 °C prior to addition. The reaction mixture was incubated for 6 h at room temperature. Subsequently, the product was extracted with DCM (3 × 50 mL) and dried over anhydrous Na_2_SO_4_. The product solution in DCM was evaporated under reduced pressure to yield the 10,12-diynoyl amide, a white solid. Next, under N_2_, the 10,12-diynoyl amide (1 equiv.) was dissolved in 140 mL of diethyl ether followed by addition of lithium aluminum hydride (12 equiv.) at 0 °C. The reaction was allowed to proceed for 20 h at room temperature. Upon reaction completion, the mixture was cooled to 0 °C, then treated with dropwise addition of water and aqueous solution of NaOH (15% w/w), then stirred for 30 min to neutralize the LiAlH_4_. Subsequently, additional water was added to the mixture, and the product solution was separated. Finally, the extracted product solution was dried over anhydrous Na_2_SO_4_ before being filtered and evaporated under reduced pressure to yield the final product. See Figure S27 for ^1^H NMR spectrum

**Peltier plate assembly design and assembly.** A Peltier plate apparatus was designed and fabricated, enabling DA polymerization on HOPG to be carried out at desired setpoint temperatures, in an inert environment. Sample setpoint temperature was maintained using a Peltier cold plate cooler (CP-031HT, TE Technologies Inc., Traverse City, MI), providing efficient direct cooling or heating from 0–75 °C. The setpoint is input using a thermometric temperature controller (TC-48-20, TE Technologies Inc., Traverse City, MI); a thermistor continuously monitors the temperature at the sample plate. Four 3D printed custom support members maintain the position of the Peltier plate above the optical breadboard, for efficient thermal dissipation from the fan to surroundings. A UV lamp (UVP-95-0201-01, Capitol Scientific, Austin, TX) is independently positioned at a fixed height above the Peltier plate, using additional custom 3D-printed supports. To achieve a photon flux equivalent to that used in our previous work (~6900 photons nm^-2^ h^-1^ , or ~2 photons nm^-2^ s^-1^), the UV lamp was mounted *ca*. 2 cm above the surface of the cold plate. Photon flux was quantified using a TR-74UI-S UV data logger (MicroDAQ, Concord, NH). The apparatus was housed in an environmental chamber (818-GB, Plas-Labs, Lansing, MI), with an N_2_ feed used to control the gas-phase composition during polymerization, and an Onset HOBO MX1101 Bluetooth Temperature and Humidity Data Logger to monitor humidity levels.

**Langmuir-Schaefer transfer to generate striped diacetylene films.** Striped phase diacetylene (DA) films were prepared based on a Langmuir-Schaefer conversion method we have reported previously^4-6^ with minor modifications, described briefly here. Langmuir-Schaefer transfers were performed using a microTrough XL Langmuir-Blodgett trough (Kibron Inc., Helsinki, Finland) with a customized temperature-controlled transfer stage. Prior to use, troughs were cleaned with ethanol followed by Milli-Q water (×3). HOPG substrates were cleaved immediately prior to sample deposition. Substrate setpoint temperature was maintained at 35 °C. This temperature is somewhat lower than we have typically used to maximize domain size, but high enough to avoid subphase condensation on the HOPG as it approaches the Langmuir film, and low enough to avoid thermal DA polymerization. For the LS transfer, 0.75 mg/mL of amphiphile in CHCl_3_ were prepared and then deposited across the surface of the aqueous subphase as 1-µL droplets. The system was allowed to equilibrate for 15 min prior to compression, which was carried out by sweeping the moveable barriers inward until the target mean molecular area (typically 30 Å^2^/chain) was achieved. Freshly cleaved HOPG substrates were mounted on the transfer stage, oriented nearly parallel to the air–water interface, and brought into contact with the subphase at a rate of 2 mm/min using the automated dipper. Following the specified contact time with the subphase, the substrates were lifted out of contact at the same rate, then blown dry with UHP N_2_. DA monolayers were polymerized between 0 – 60 min by placing them on the Peltier plate at the desired set-point temperature before irradiated under a UV lamp (λ_max_ = 254 nm, 8 W), with ~2 cm between the lamp and the substrate. To ensure equivalent photon flux, this parameter was measured at several locations under the lamp, and samples were placed in locations with equivalent flux. Respective trough parameters for TCD-COOH, TCD-OH, and TCD-NH_2_ can be found in the table below.

Table S1. Langmuir film preparation parameters for each amphiphile.

| **amphiphile** | **vol deposited (µL)** | **subphase** | **substrate‒subphase contact time (min)** | **barrier speed (mm/min)** | **target pressure (mN/m)** |
| --- | --- | --- | --- | --- | --- |
| 10,12-tricosadiynoic acid | 34.5 | Milli-Q water (~ 18 MΩ · cm) | 1 min | 2.55 | ~4-5 |
| 10,12-tricosadiyn-1-ol | 34.5 | Milli-Q water (~ 18 MΩ · cm) | 2 min | 6.00 | ~6-8 |
| 10,12-tricosaalkyldiynamine | 33.0 | 40 mM CaCl_2_ | 2 min | 6.00 | ~10-15 |

**DSC thermal characterization for TCD-COOH, TCD-OH, and TCD-NH_2_.** Measurements were performed with a Perkin Elmer Jade DSC for samples of amphiphiles with and without graphene nanoplatelets (GNP). *Sample preparation steps*: First, the amphiphile was removed from the freezer and equilibrated to room temperature (~30 min). Samples were then dissolved in CHCl_3_ and filtered with a 25-mm PTFE syringe filter to remove any oligomers; subsequently, amphiphiles were dried under UHP N_2_ (approximately 30-45 min), under vacuum if necessary. Samples of amphiphiles without GNP (-GNP) were weighed directly into an aluminum pan and characterized by DSC. *For samples with GNP (+GNP)*: First, ~10 mg of GNP were weighed and placed in a heated vacuum oven (Fisher Scientific Isotemp Vacuum Oven Model 281A) for 24 h at ~250 °C under -30 in. Hg vacuum to promote removal of adsorbates that could affect adsorption of amphiphiles. Based on the calculated total surface area of weighed GNP (using vendor-specified specific surface area SA=130 m^2^g^-1^), the theoretical amount of amphiphile needed to generate at least 10 layers was calculated and prepared in 0.5 mL of CHCl_3_. Next, the GNP were mixed with amphiphile solution and gently shaken by hand for 30 s, before drying under UHP N_2_ (30‒45 min). Subsequently, prepared amphiphiles with GNP were weighed into an aluminum pan and characterized by DSC. To ensure detection of phase transitions, approximately 4.5–5.5 mg of material was used in each sample. The following DSC temperature ramps were utilized:

1. Hold for 1 min at 25 °C
2. Heat from 25 °C to 120 °C @ 10 °C/min
3. Hold for 1 min at 120 °C
4. Cool from 120 °C to 0 °C @ 0.5 °C/min
5. Hold for 3 min at 0 °C
6. Heat from 0 °C to 120 °C @ 2.0 °C/min

**Evaluation of melting transition with temperature-controlled atomic force microscopy (AFM) imaging.** Prepared monolayers were imaged using a Cypher ES AFM (Oxford Instruments, Santa Barbara, CA). The setpoint of the temperature-controlled stage was varied from 25–85 °C, using AC Air Topography mode, with images acquired at 10 °C increments. A 0.01‒0.02 °C/s temperature ramp was used when the setpoint was changed, to avoid AFM tip collisions with the surface due to thermal drift. Additionally, to closely monitor surface changes with temperature, tip contact was often maintained during the temperature ramp. Cantilever oscillation and calibration were carried out utilizing blueDrive photothermal excitation to maintain stable, high-resolution images. Phase shift values of 90° and above were maintained (attractive interaction regime), to minimize monolayer restructuring due to surface contact. Samples subjected to one heating cycle were not used for subsequent thermal imaging cycles, to ensure observed melting transitions were not altered by monolayer annealing in a prior heating/imaging cycle. Imaging was carried out using Bruker RFESP-75 tips (nominal force constant 3 N/m and radius of curvature <12 nm).

**Determining optimal polymerization temperature conditions for TCD-NH_2_ and TCD-OH.** To establish optimal polymerization temperature conditions for TCD-NH_2_ and TCD-OH, we quantified transfer efficiency to PDMS from 25–75 °C for conversion of monomers after 60 min of photopolymerization time. All photopolymerization reactions were performed in an environmental chamber flooded with UHP N_2_ until relative humidity levels reached 10 % or lower, to ensure an inert environment. Utilizing the Peltier plate assembly described above, substrates were positioned on the plate at locations selected to ensure uniform UV intensity. Samples were equilibrated at the set-point temperature for 15 min and then irradiated with a UV lamp mounted ~2 cm above the surface of the Peltier plate while maintaining set temperature conditions. After the desired polymerization time, samples were removed from the UV-illuminated area and stored at room temperature under UV-free conditions prior to transfer to PDMS.

**Covalent transfer of sPDA layers from HOPG to PDMS.** Transfer of sPDA layers from HOPG to PDMS was carried out using a minor adaption of our previously reported protocol.^7^ Sylgard 184 silicone base and curing (crosslinking) agent were mixed in a 10:1 (*mass/mass*) ratio. After components were thoroughly mixed with a stir bar at 200 rpm (~10 min), the mixture was poured over HOPG substrates functionalized with sPDA monolayers. The PDMS-coated substrates were placed in a vacuum chamber for at least 30 min, or until no bubbles were observed. PDMS-coated substrates were subsequently cured at a relatively low temperature (35 °C) for 24 h to avoid thermal polymerization of PDAs (see further discussion of condition optimization, in a later section of the Supporting Information). The cured PDMS was then gently exfoliated from the HOPG and stored under ambient conditions prior to further characterization.

**Confocal fluorescence microscopy and spectral imaging.** Fluorescence images and emission spectra were acquired using a Zeiss LSM 880 Axio Examiner upright confocal microscope. Functionalized PDMS samples were imaged with a 20× objective (plan-apochromatic, dry, NA = 0.80, no coverslip) immersed in water. Image excitation was carried out using a 488-nm Ar laser at 100% power. Emitted fluorescence was detected by a 32-channel GaAsP spectral photomultiplier detector with a pinhole size set to 1 Airy unit. All fluorescence images and corresponding spectra were collected at a resolution of 2856 × 718 pixels with 8-bit depth, 875 gain. Unidirectional horizontal scans were averaged 4 times/line with a dwell time of 3.77 μs/pixel. Emission spectra were collected from 495–691 nm with a bin width of 8.9 nm when evaluating the PDA transfer.

**Discussion of experimental observables related to alkyl chain disordering in bulk vs monolayer diacetylene assemblies.** In bulk diacetylene assemblies (crystals or multilayer films), chromism is understood as a spectroscopic signature of the conjugated PDA backbone conformation: the “blue phase” corresponds to a relatively planar, extended backbone, whereas the “red phase” reflects increased torsion and reduced effective conjugation length. Thermochromism, in turn, tracks temperature-dependent shifts in this torsional landscape, which are strongly coupled to ordering transitions of the surrounding alkyl chains. In practice, these effects are typically quantified in 3D crystals, multilayer films, or micelles by UV–vis absorption, where large extinction coefficients for PDAs enable clear readouts of phase state and transition temperatures in samples with micrometer-scale optical path lengths.

For monolayers on solid substrates, such as those measured here, this conventional optical readout is not accessible. For instance, utilizing absorbance values from a prior publication aimed at determining polymer content in nonsoluble PDA films,^8^ a 35-nm thick standing phase film of 10,12-pentacosadiynoic acid (the 25-carbon analogue of 23-carbon 10,12-tricosadiynoic acid used in the present work) yielded red and blue phase absorbance maxima from 0.10 (red) to 0.15 (blue). Therefore the 0.5-nm thick films used here would produce absorbance values lower by a factor of 70: 0.0014 (red) and 0.0021 (blue), which are well outside commonly accepted measurement ranges. Fluorescence is also not a reliable alternative for monolayers on HOPG; as we have reported previously,^7^ even for monolayer that exhibit fluorescence once transferred to PDMS, we do not observe fluorescence on HOPG, consistent with strong substrate-mediated quenching and/or ‘blue’-phase backbone conformation at the interface.

Instead, the present measurements probe the structural *correlates* of chromism and thermochromism at the interface. Our approach is conceptually analogous to comparing bulk melting with surface melting in DSC: rather than measuring a bulk optical response, we quantify temperature-dependent disorder within a single molecular layer. In this framework, increases in monolayer disorder, particularly within the alkyl chains, serve as a proxy for the same physical drivers that, in bulk, promote backbone twisting and the blue-to-red transition. Importantly, interactions between the alkyl chains and the HOPG substrate shift these disordering transitions to higher temperatures relative to bulk, potentially also impacting backbone behavior.

**Molecular models of TCD-COOH, TCD-OH and TCD-NH_2_ dynamics at temperatures from 25‒95 °C.**

We carried out molecular dynamics simulations to compare monolayer stability across a range of temperatures relevant to on-surface diacetylene polymerization. Model systems were generated using Schrodinger Maestro, and dynamics were carried out using the Desmond software package within the Schrodinger interface. Desmond generates a user-specified parallelepiped box around the model system; this global cell is used to establish periodic boundary conditions for dynamics.

Initial models of TCD-COOH, TCD-OH, and TCD-NH_2_ monolayers were created by generating 4 rows of 34 molecules each on a layer of graphene, with 8 pairs of molecules removed from the left pair of molecular rows as shown in Figure S1. In the initial model, prior to relaxation, molecules are slightly overpacked, to ensure they do not overhang the edge of the graphite sheet, the dimensions of which are used to ensure consistency of the global cell parameters. The Desmond System Builder utility was used to create a molecular dynamics input model using no solvent, box size buffer parameters of 0.1 Å at left and right edges of the model (in the orientation shown in the figure), 0.2 Å buffer at the top and bottom edges, and 10 Å buffer perpendicular to the image plane. These parameters were chosen to create a unit cell that nearly approximates the behavior of a monolayer on a large sheet of graphite. The 0.2 Å buffer at the top and bottom edges was required to accommodate the aligned placements of terminating hydrogens at the top and bottom edges of the model; hydrogens on the left and right edges are staggered, permitting use of the 0 Å buffer. These models were used as inputs to Desmond Molecular Dynamics runs, with a typical simulation time of 10 ns, 1.2 ps energy calculation steps and 5 ps trajectory recording steps, the NVT ensemble class, and a specified temperature from 298 to 368 K. Simulations were carried out with the graphene sheet constrained with a force constant of 100 kJ/mol Å. As the system is relaxed prior to dynamics, the molecules expand through the connected left and right edges of the box, as shown in Figure S1b; alkyl chain segments that appear to be freestanding are in contact with the opposing edge of the graphene sheet, through the box edge. The packing density shown creates alkyl chain spacings of ~4.7 Å, similar to spacings reported previously based on STM experiments by others.^9^


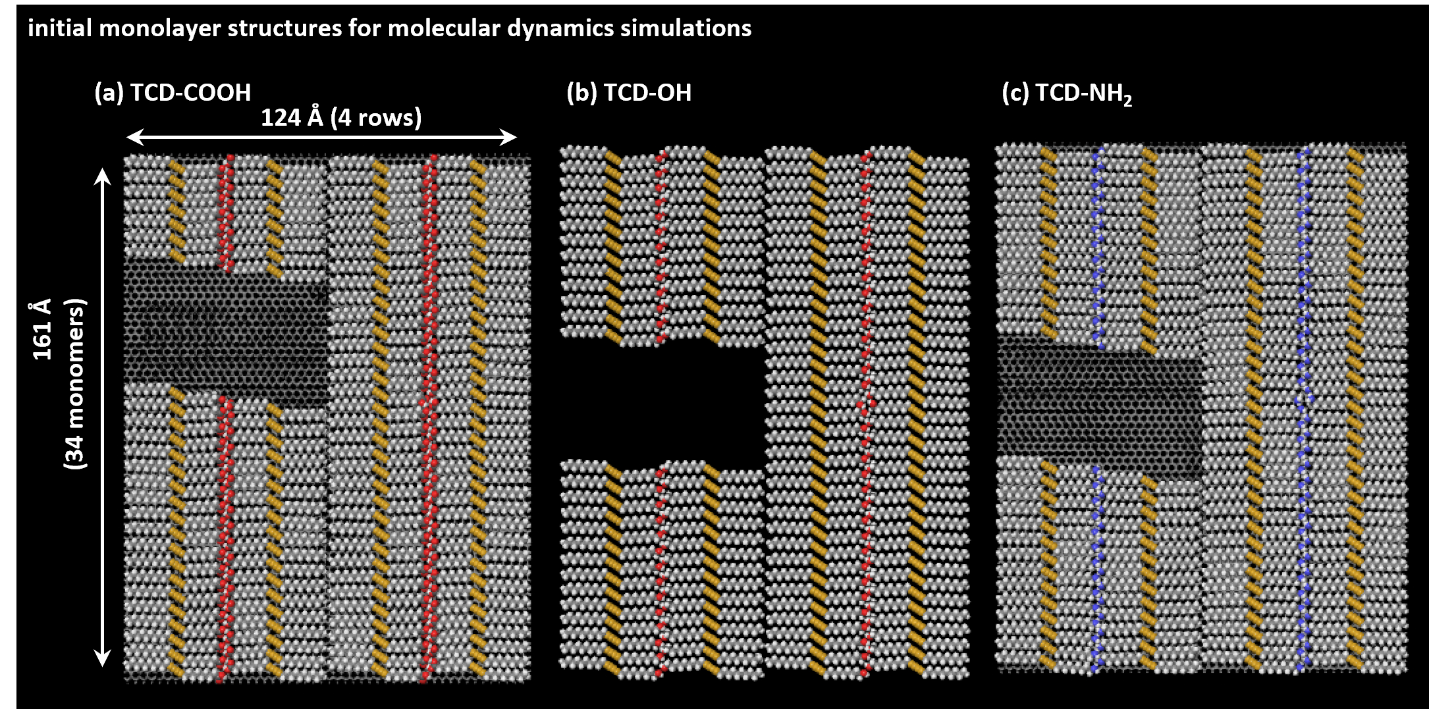


**Figure S1.** Initial molecular models of (a) TCD-COOH, (b) TCD-OH, and (c) TCD-NH_2_ monolayers on graphene sheets, prior to relaxation with 8 pairs of molecules removed from the left two rows to allow for molecular disordering with temperature.

Figures S2‒S4 show representative snapshots from the end of each 10 ns dynamics simulation, to facilitate visual comparison of monolayer stability across the tested temperature range. In TCD-COOH models (Figure S21), only a few alkyl chains near the end of the vacancy rows exhibit disorder after 10 ns. At 85 °C, molecules in the vacancy rows tilt, and at 95 °C, the vacancy rows are disordered. The fully-packed TCD-COOH rows remain ordered with the exception of one pair of molecules that slips laterally relative to the lamellar axis. Notably, the COOH dimers formed between opposing headgroups are strong enough that molecules typically remain paired even as the lamellae in the vacancy rows become disordered.

Paired headgroups in TCD-OH models (Figure S3) also exhibit reasonably strong H-bonding. However, unlike COOH pairs, the H-bonding interaction is not optimized with the molecular axes perpendicular to the lamellar axis, leading to tilting of the molecular axes even at fairly low temperatures, as has been observed previously in STM experiments by others.^10^ At 85 °C, more substantial molecular disorder becomes evident, and at 95 °C, all molecular rows have disordered within 10 ns. H-bonding between paired TCD-NH_2_ headgroups is weaker (Figure S4), and moderate disordering is observed in the vacancy lamellae at 55 °C, with significant disorder at 75 °C. Defects often involve breaking H-bonds between headgroups. Sets of atoms were selected and their radial distribution functions and respective integrals calculated for the period from 5 to 10 ns, as described in the main manuscript.

**
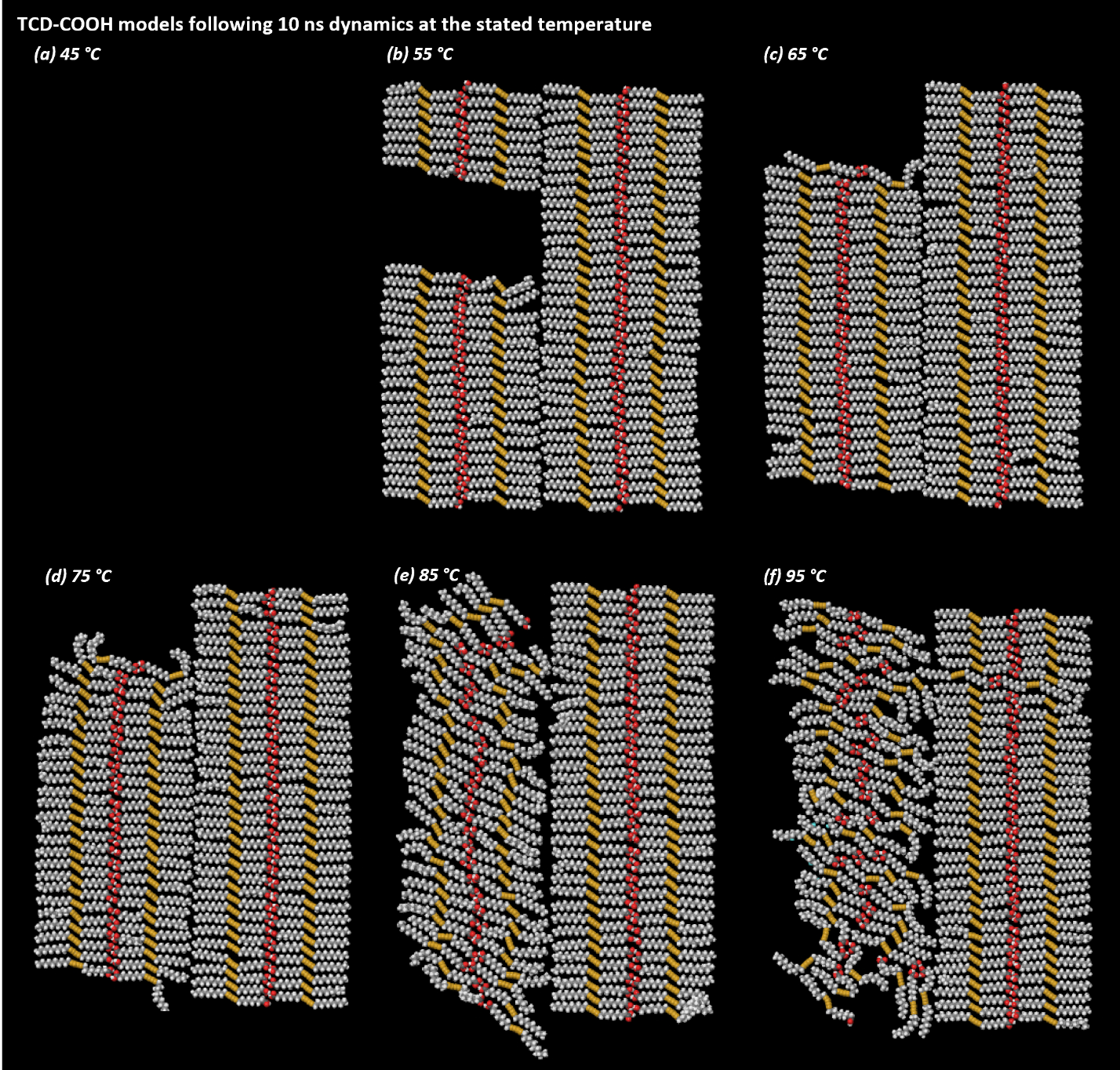
**

**Figure S2.** Molecular model of TCD-COOH monolayer on graphene sheet with 34 molecules per row (4.7 Å alkyl chain repeat distance) and 8 pairs of molecules removed from left two rows, after dynamics carried out for 10 ns at (a) 45 °C, (b) 55 °C, (c) 65 °C, (d) 75 °C, (e) 85 °C, and (f) 95 °C.


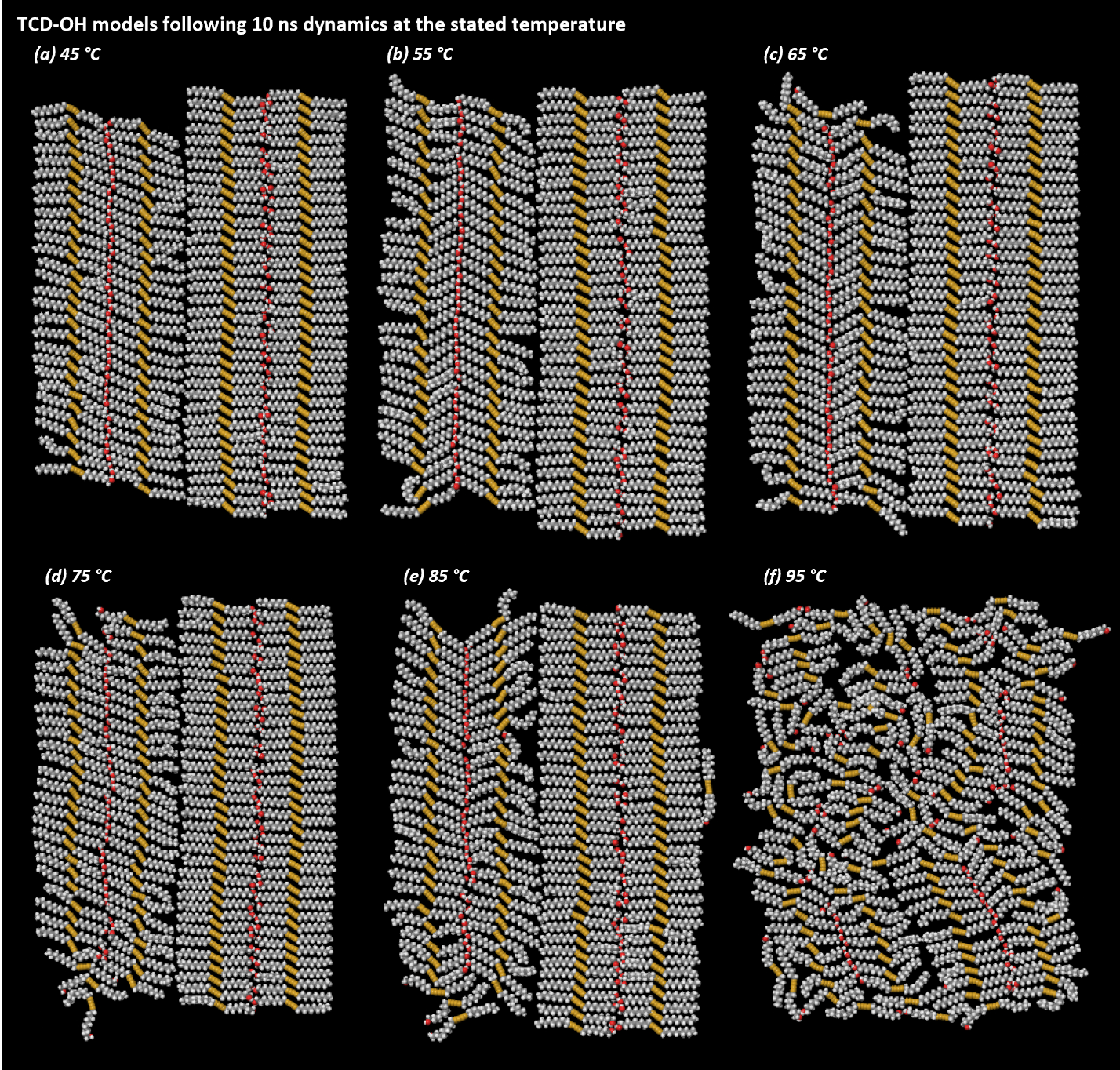


**Figure S3.** Molecular model of TCD-OH monolayer on graphene sheet with 34 molecules per row (4.7 Å alkyl chain repeat distance) and 8 pairs of molecules removed from left two rows, after dynamics carried out for 10 ns at (a) 45 °C, (b) 55 °C, (c) 65 °C, (d) 75 °C, (e) 85 °C, and (f) 95 °C.


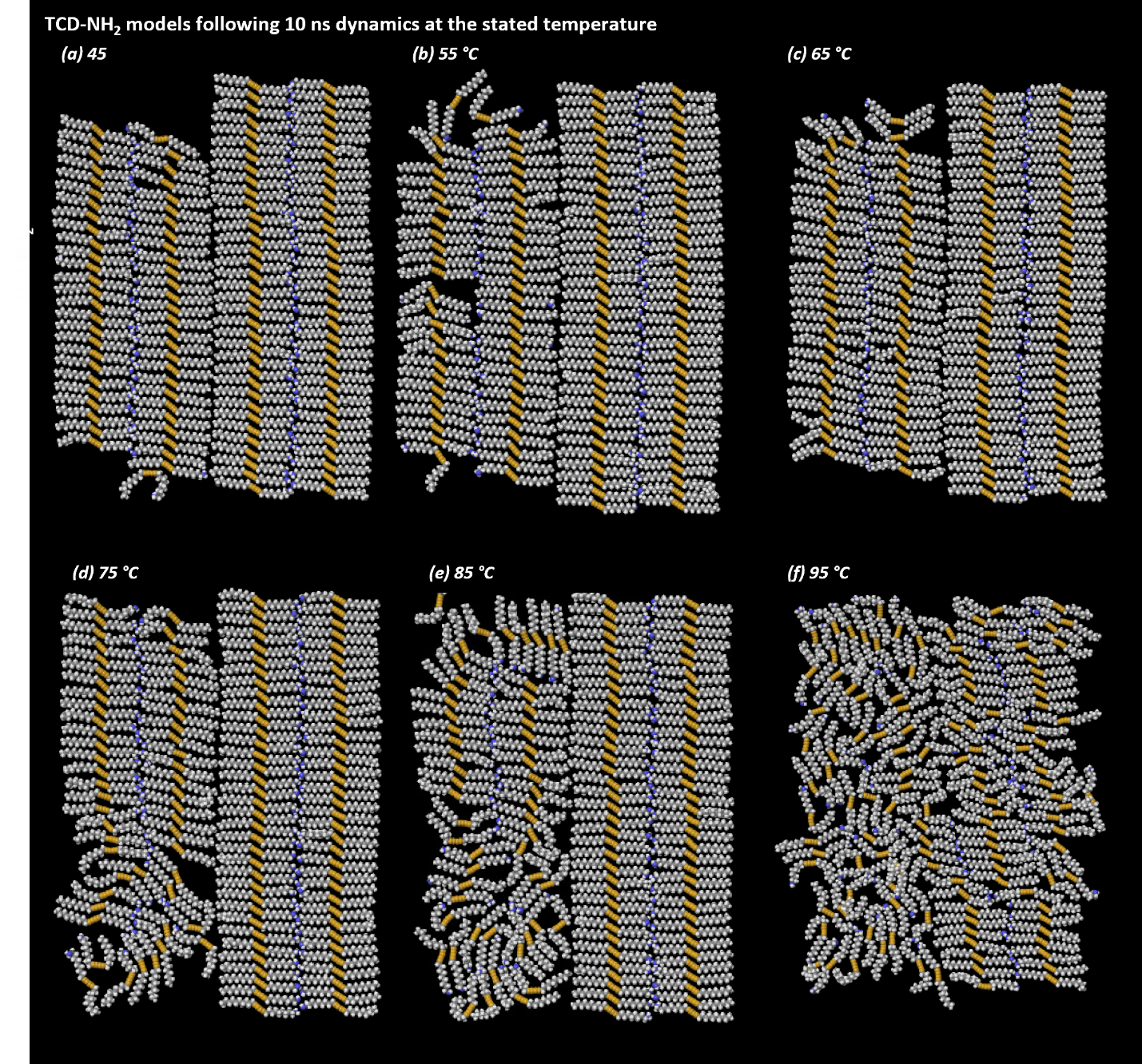


**Figure S4.** Molecular model of TCD-NH_2_ monolayer on graphene sheet with 34 molecules per row (4.7 Å alkyl chain repeat distance) and 8 pairs of molecules removed from left two rows, after dynamics carried out for 10 ns at (a) 45 °C, (b) 55 °C, (c) 65 °C, (d) 75 °C, (e) 85 °C, and (f) 95 °C.

**DSC thermograms for alkyldiacetylenes with and without graphene nanoplatelets.** In the main manuscript, we present DSC thermograms for the second heating cycles of TCD-COOH, TCD-OH and TCD-NH_2_ with and without graphene nanoplatelets (GNP). Here, Figures S5‒S14 present thermograms for the first heating cycle, cooling cycle, and second heating cycle for GNP and the three molecules, to facilitate visual inspection. Table S2 lists peak positions from the thermograms.


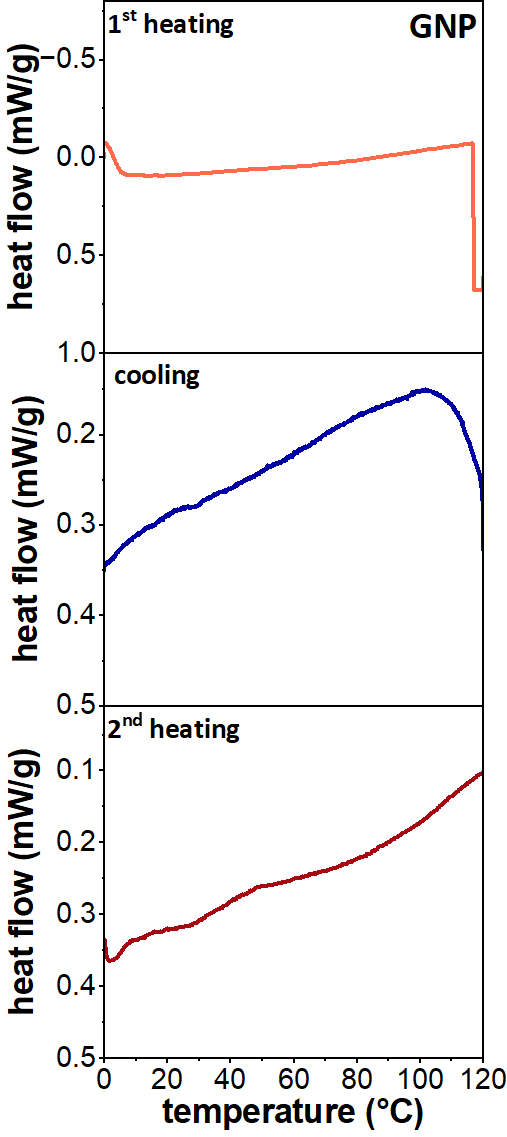


**Figure S5.** DSC thermograms for the first heating cycle (top panel), cooling cycle (middle panel), and 2^nd^ heating cycle (bottom panel) of graphene nanoplatelets (GNP).


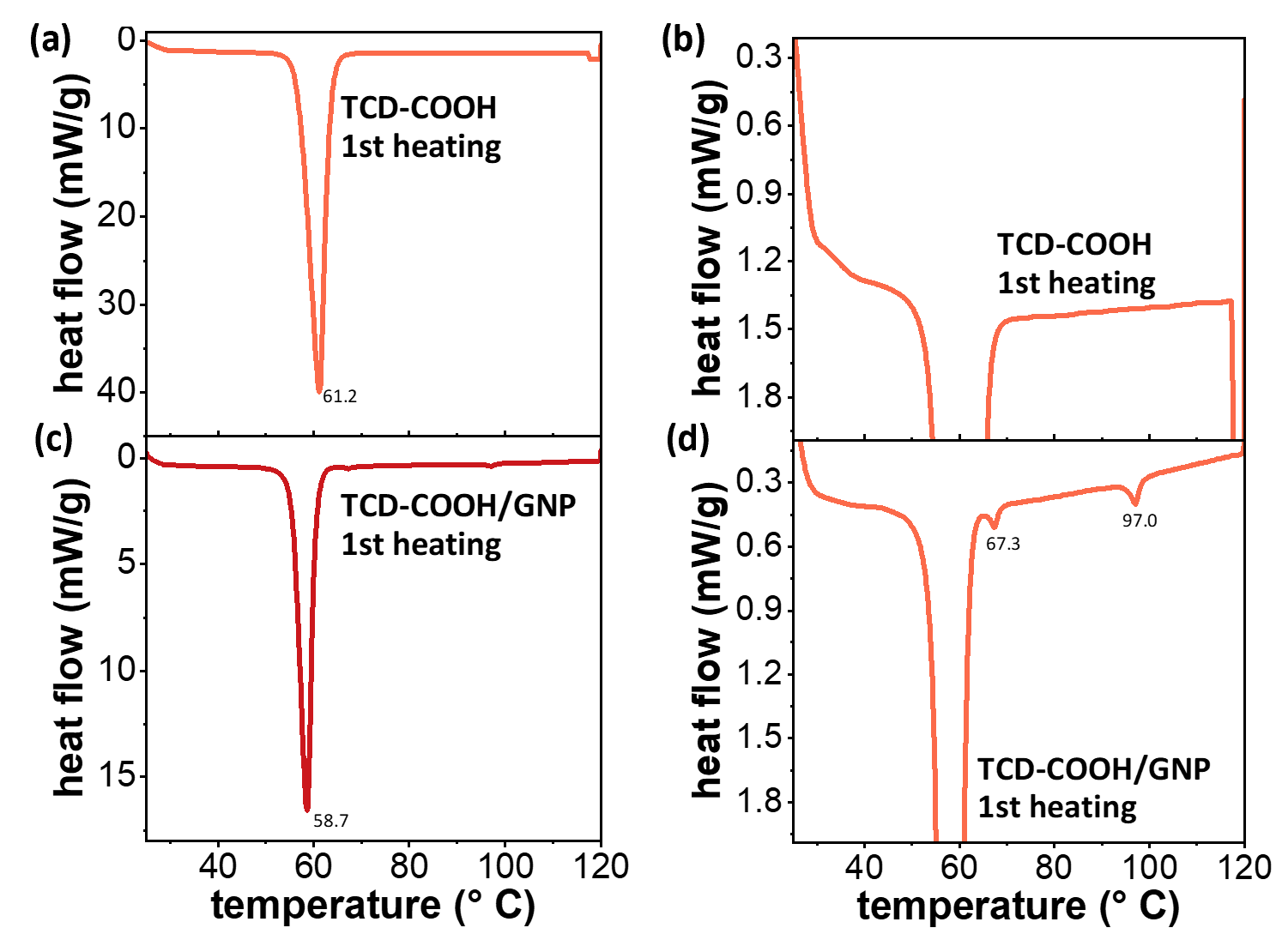


**Figure S6.** DSC thermograms of first heating cycle for TCD-COOH (a,b) without and (c,d) with graphene nanoplatelets (GNP). Panels (b) and (d) show enlargements around the baselines of the thermograms to facilitate visual inspection of small peaks such as those corresponding to thermal transitions in the layer adsorbed to the GNPs.


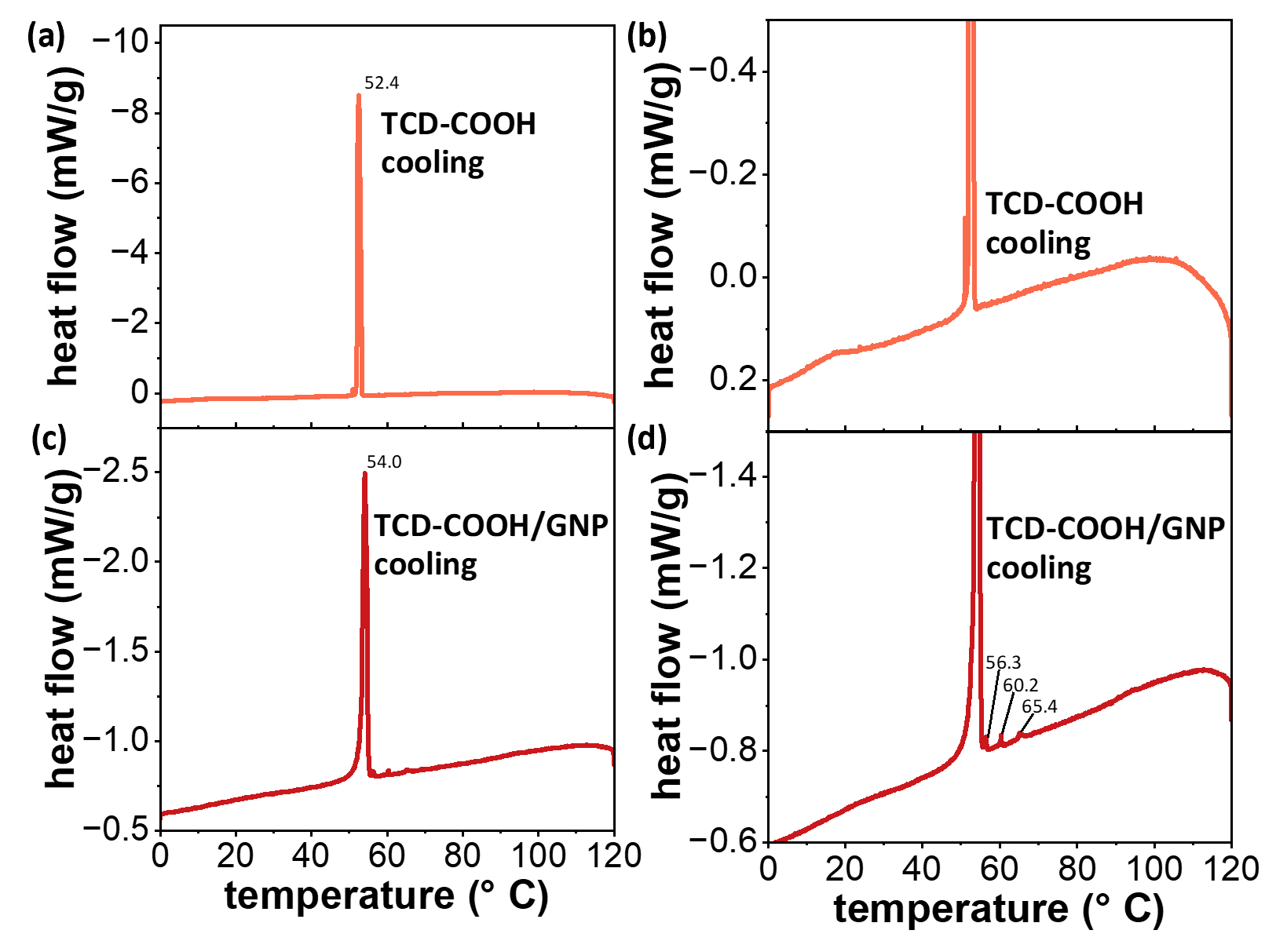


**Figure S7.** DSC thermograms of cooling cycle for TCD-COOH (a,b) without and (c,d) with graphene nanoplatelets (GNP). Panels (b) and (d) show enlargements around the baselines of the thermograms to facilitate visual inspection of small peaks such as those corresponding to thermal transitions in the layer adsorbed to the GNPs.


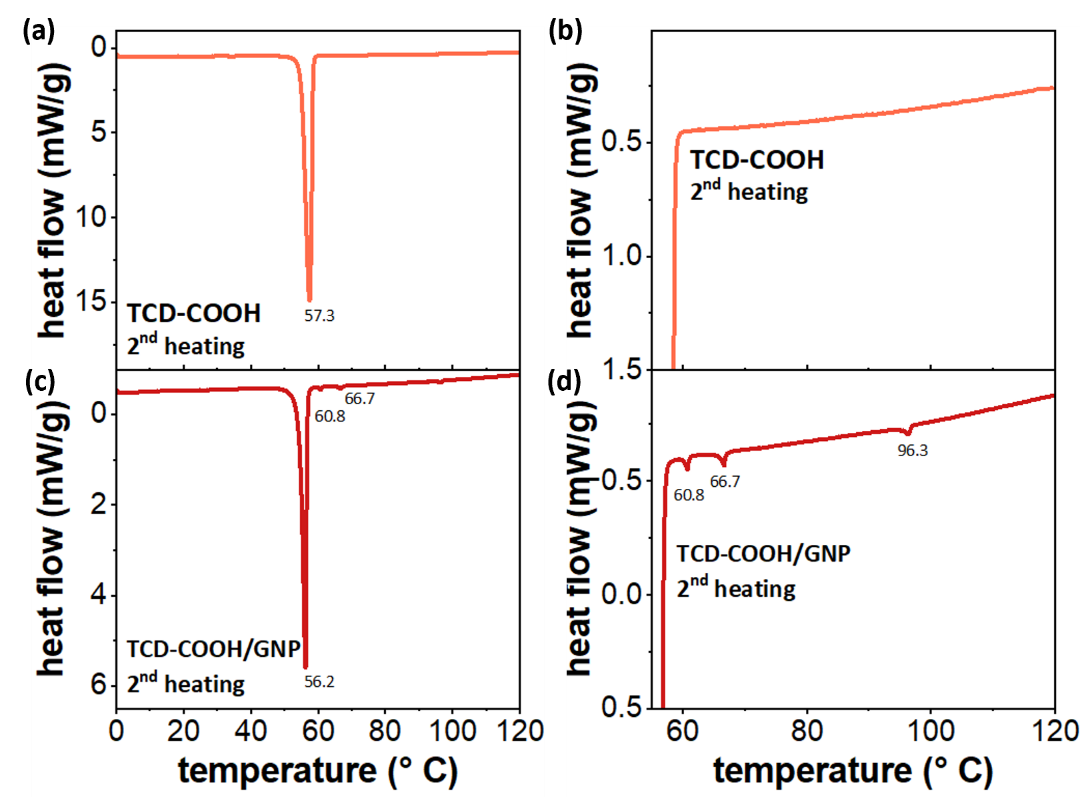


**Figure S8.** DSC thermograms of second heating cycle for TCD-COOH (a,b) without and (c,d) with graphene nanoplatelets (GNP). Panels (b) and (d) show enlargements around the baselines of the thermograms to facilitate visual inspection of small peaks such as those corresponding to thermal transitions in the layer adsorbed to the GNPs.


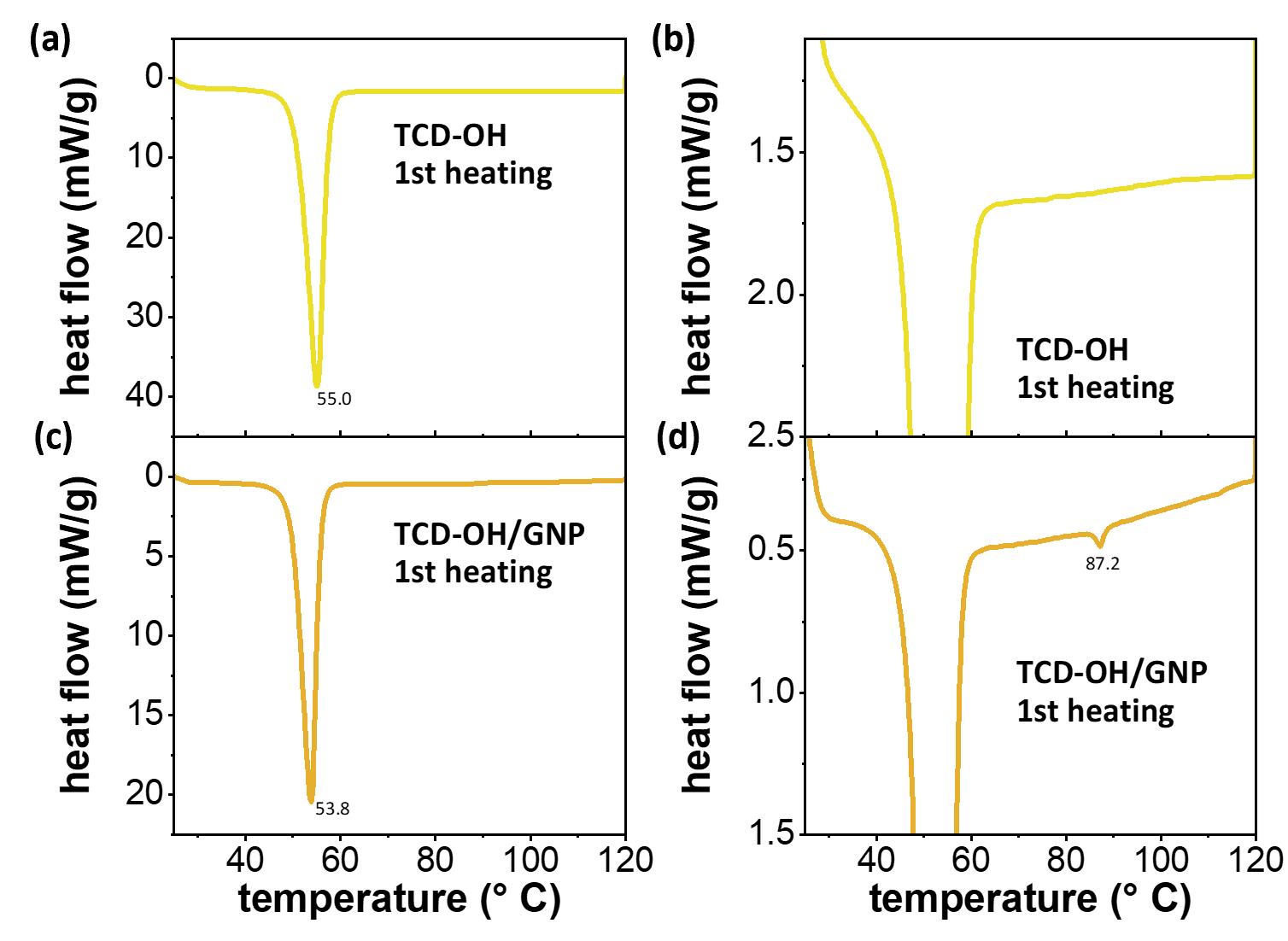


**Figure S9.** DSC thermograms of first heating cycle for TCD-OH (a,b) without and (c,d) with graphene nanoplatelets (GNP). Panels (b) and (d) show enlargements around the baselines of the thermograms to facilitate visual inspection of small peaks such as those corresponding to thermal transitions in the layer adsorbed to the GNPs.


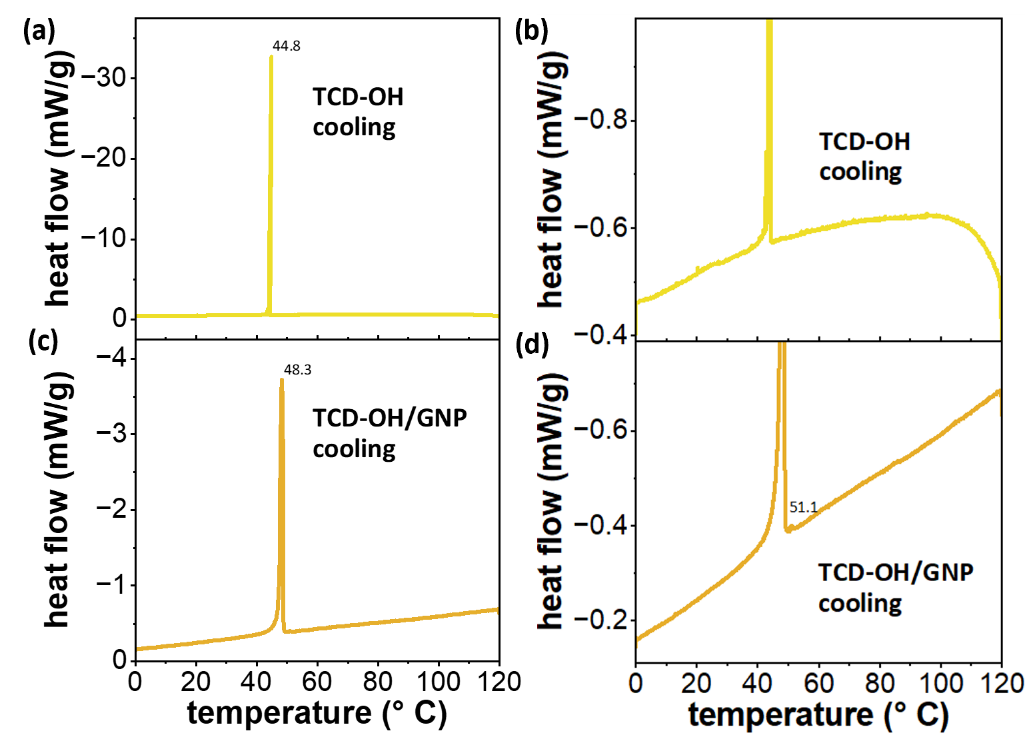


**Figure S10.** DSC thermograms of cooling cycle for TCD-OH (a,b) without and (c,d) with graphene nanoplatelets (GNP). Panels (b) and (d) show enlargements around the baselines of the thermograms to facilitate visual inspection of small peaks such as those corresponding to thermal transitions in the layer adsorbed to the GNPs.


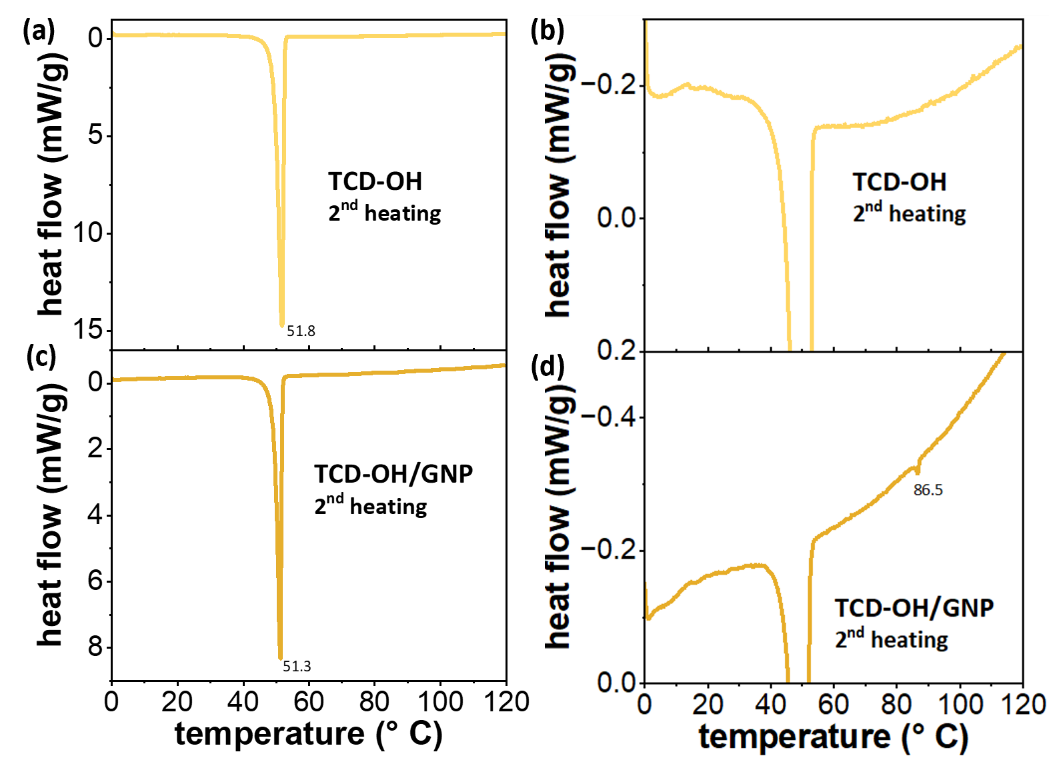


**Figure S11.** DSC thermograms of second heating cycle for TCD-OH (a,b) without and (c,d) with graphene nanoplatelets (GNP). Panels (b) and (d) show enlargements around the baselines of the thermograms to facilitate visual inspection of small peaks such as those corresponding to thermal transitions in the layer adsorbed to the GNPs.


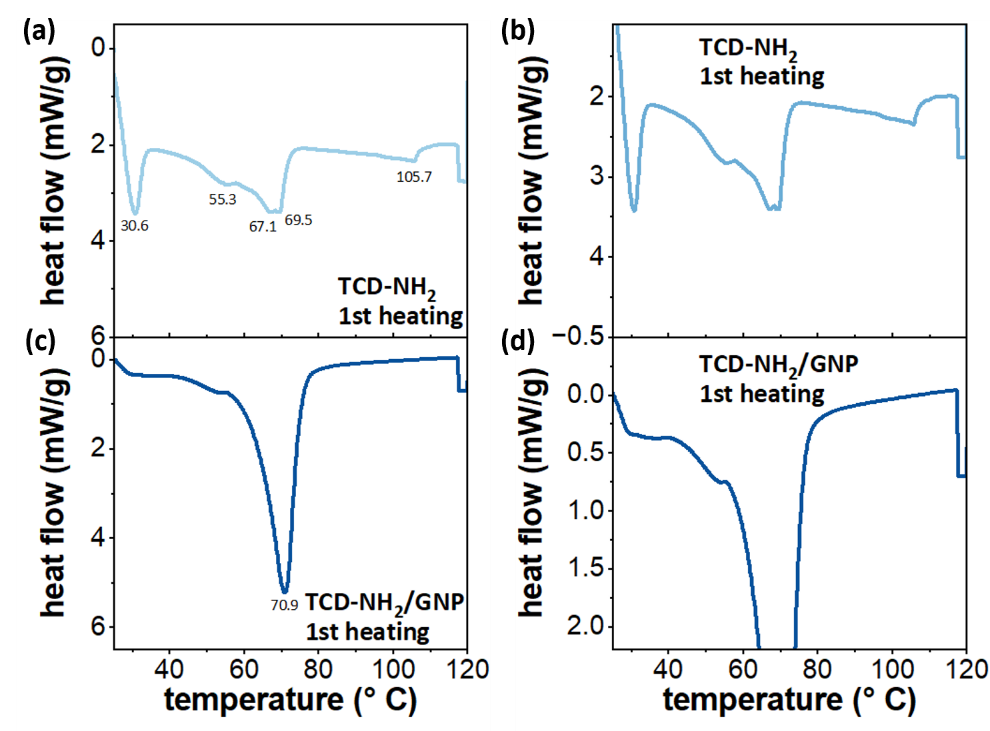


**Figure S12.** DSC thermograms of first heating cycle for TCD-NH_2_ (a,b) without and (c,d) with graphene nanoplatelets (GNP). Panels (b) and (d) show enlargements around the baselines of the thermograms to facilitate visual inspection of small peaks such as those corresponding to thermal transitions in the layer adsorbed to the GNPs.


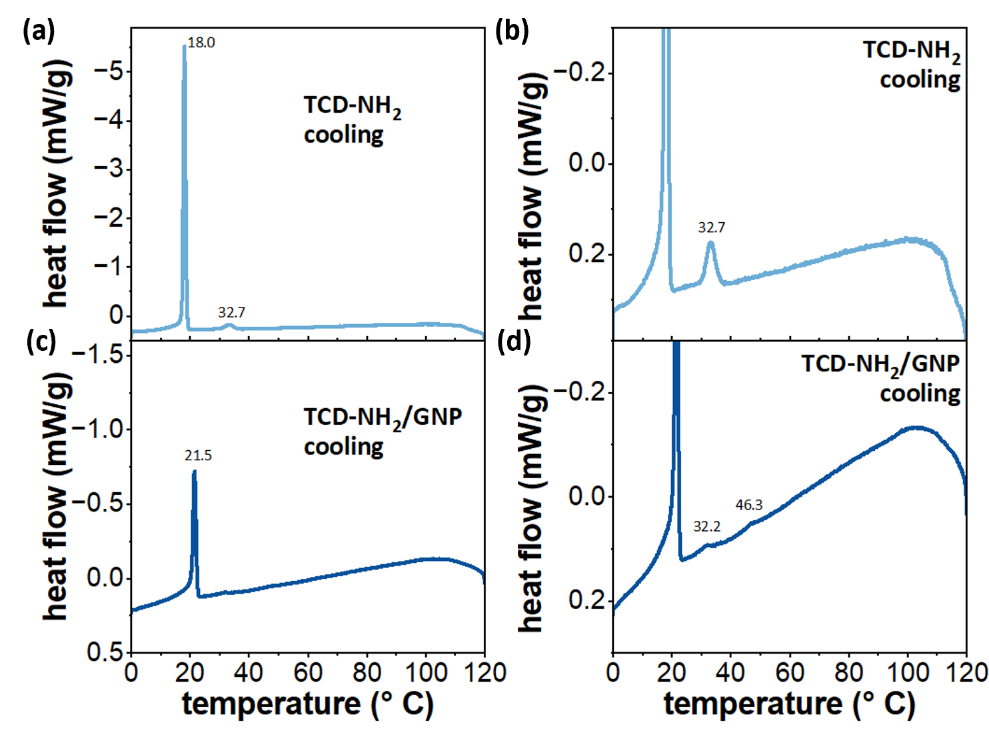


**Figure S13.** DSC thermograms of cooling cycle for TCD-NH_2_ (a,b) without and (c,d) with graphene nanoplatelets (GNP). Panels (b) and (d) show enlargements around the baselines of the thermograms to facilitate visual inspection of small peaks such as those corresponding to thermal transitions in the layer adsorbed to the GNPs.


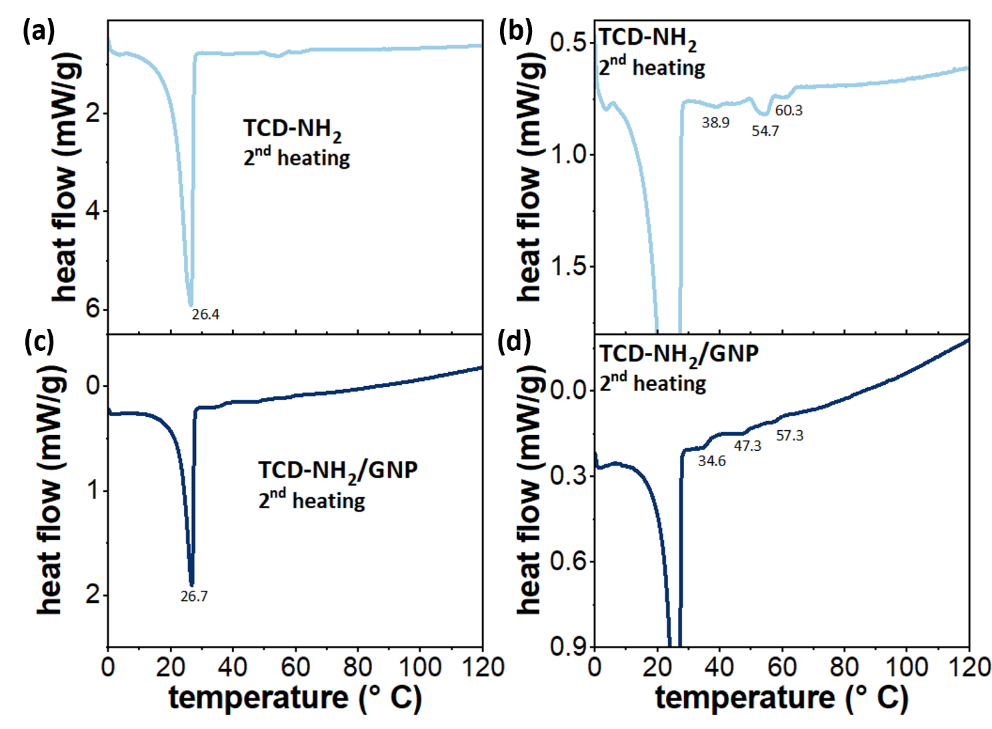


**Figure S14.** DSC thermograms of second heating cycle for TCD-NH_2_ (a,b) without and (c,d) with graphene nanoplatelets (GNP). Panels (b) and (d) show enlargements around the baselines of the thermograms to facilitate visual inspection of small peaks such as those corresponding to thermal transitions in the layer adsorbed to the GNPs.


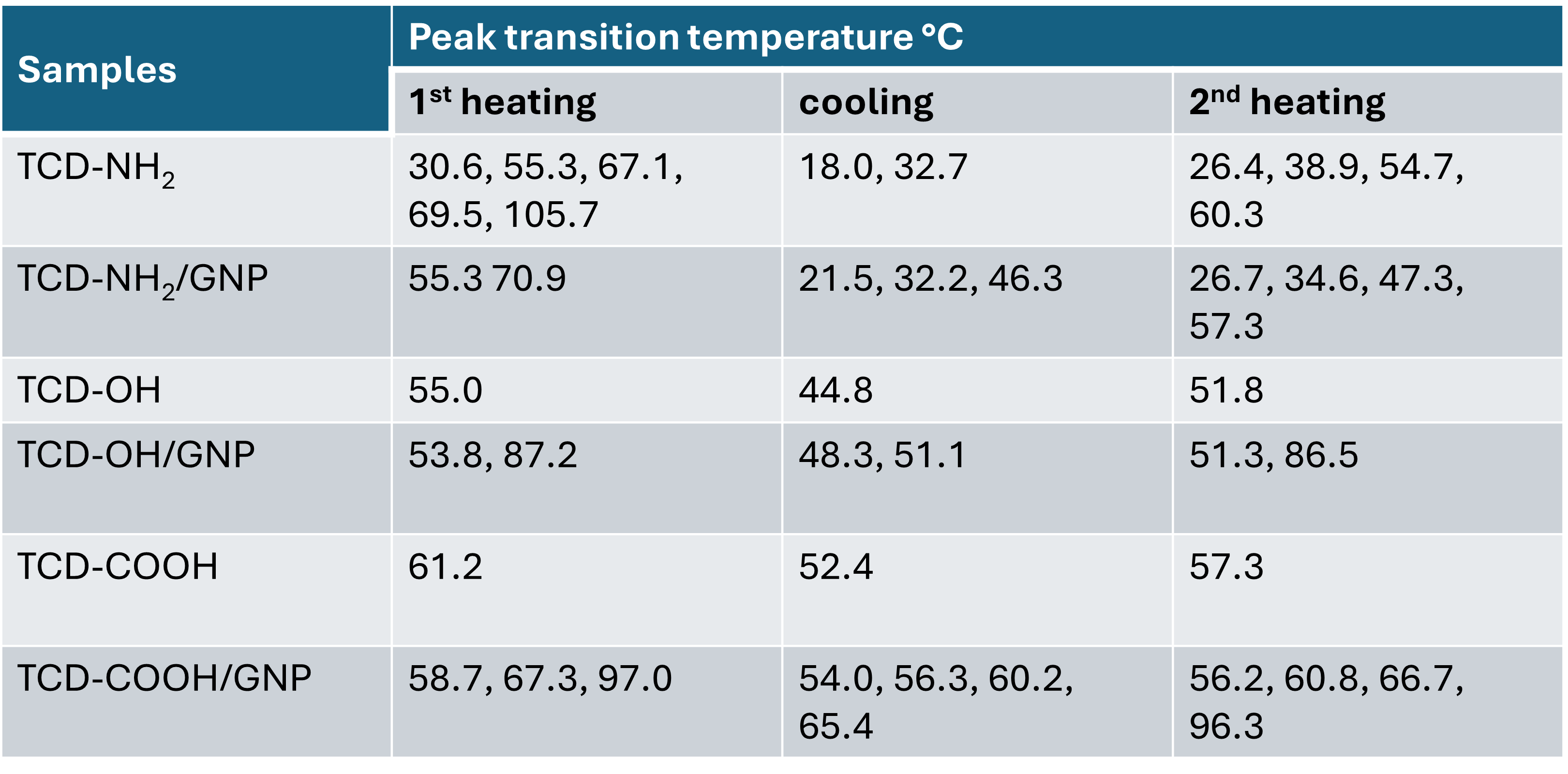


**Table S2.** Peaks from thermograms in Figures S2‒S10.

**AFM image analysis.** AFM images were processed with Gwyddion SPM software (<http://gwyddion.net>) and ImageJ (https://imagej.net/ij/download.html). Prior to quantitative analysis, mean plane subtraction and row alignment procedures, such as fitting to median or median differences, were performed for all raw data files in Gwyddion.


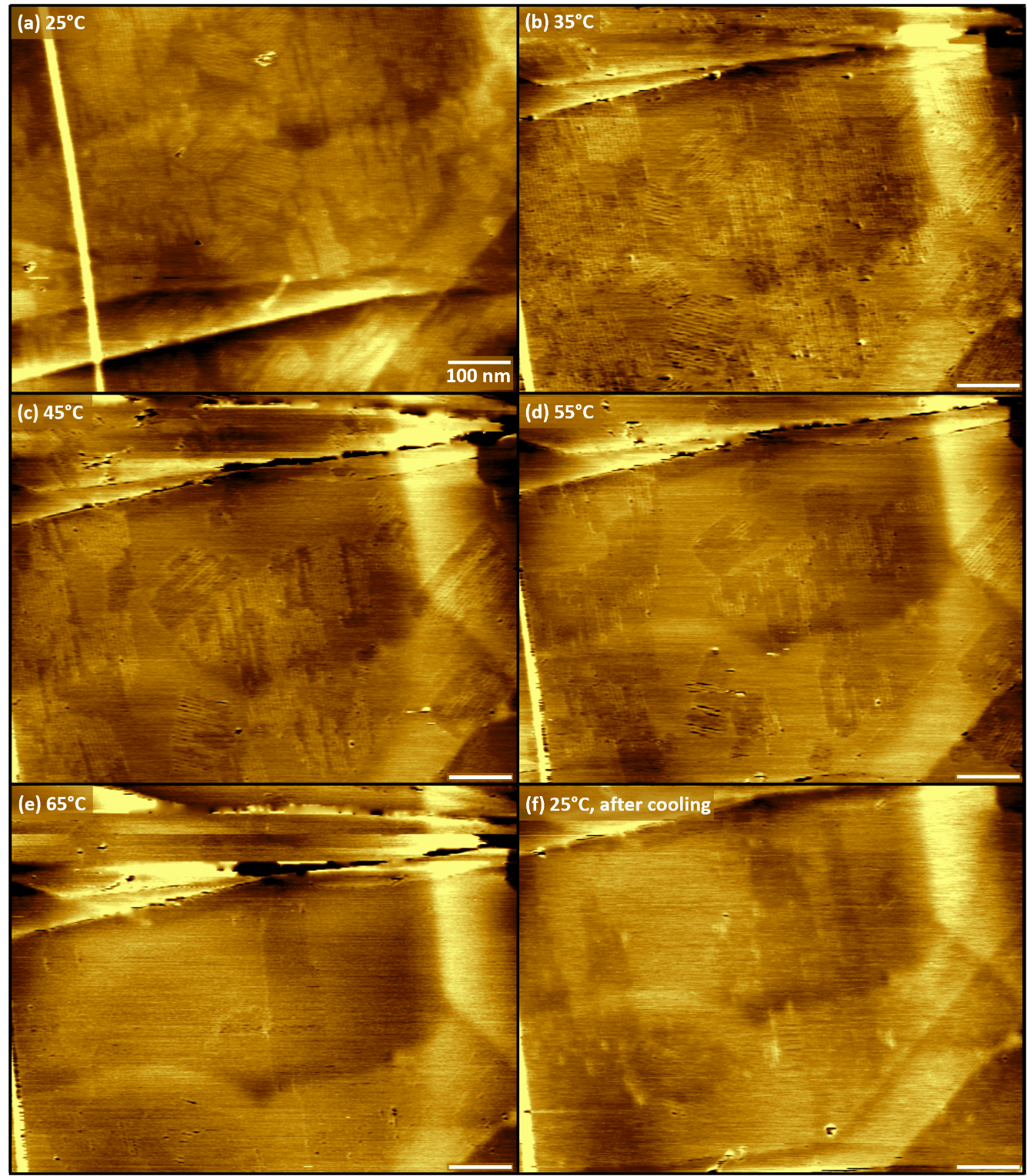


**Figure S15.** AFM height images of unpolymerized TCD-NH_2_ on HOPG, including larger areas around the region shown in Figure 5 of the main manuscript. Images were acquired at 25 °C (a), 35 °C (b), 45 °C (c), 55 °C (d), 65 °C (e), and 25 °C (f) after steady cooling from 65 °C.


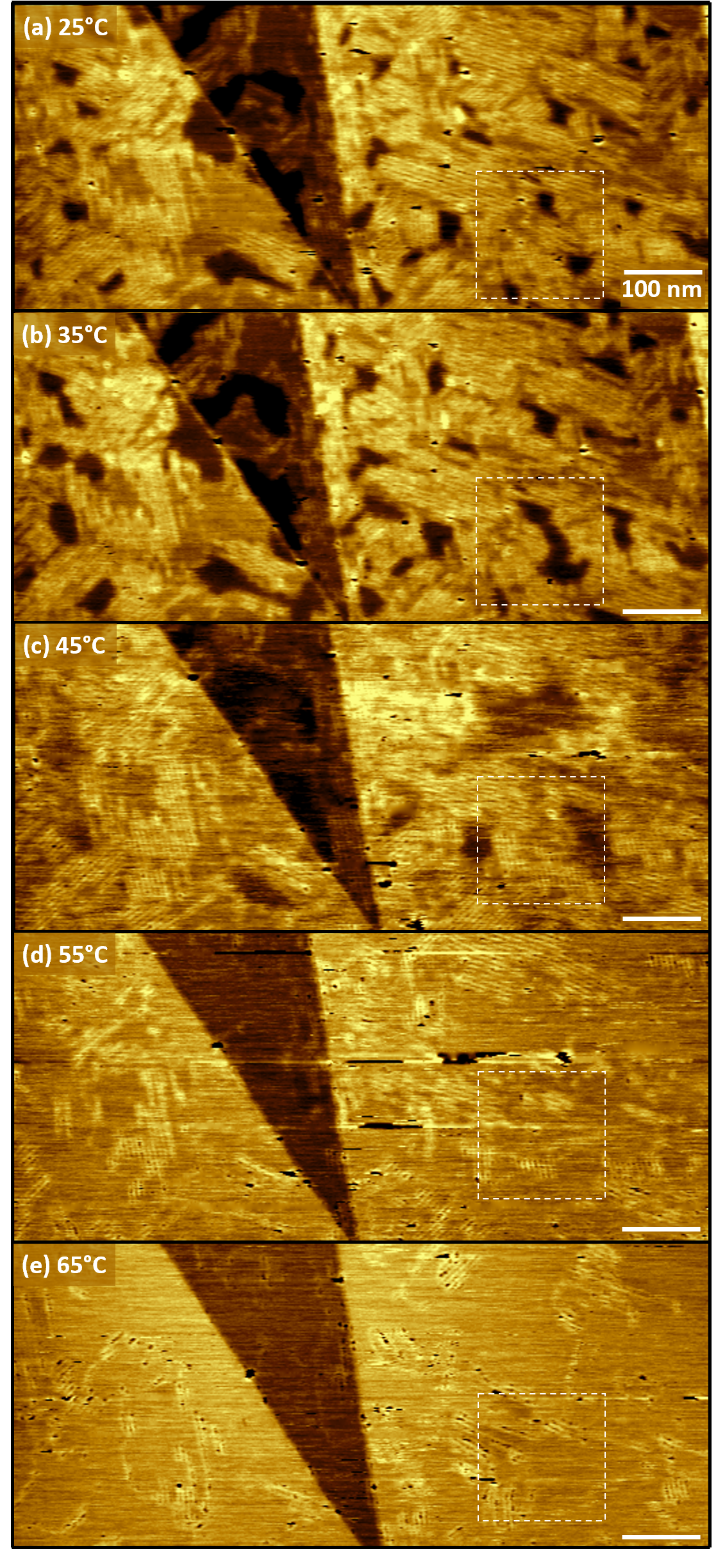


**Figure S16.** AFM height images of partially polymerized TCD-NH_2_ monolayer on HOPG prepared at PT25 °C irradiated with UV light for 20 min. Above images were acquired at 25 °C (a), 35 °C (b), 45 °C (c), 55 °C (d), and 65 °C (e). The white dashed box depicts one of several regions that demonstrate large molecular rearrangement with increasing temperature, which are observed based on variation in the shape and area of adjacent vacant regions.


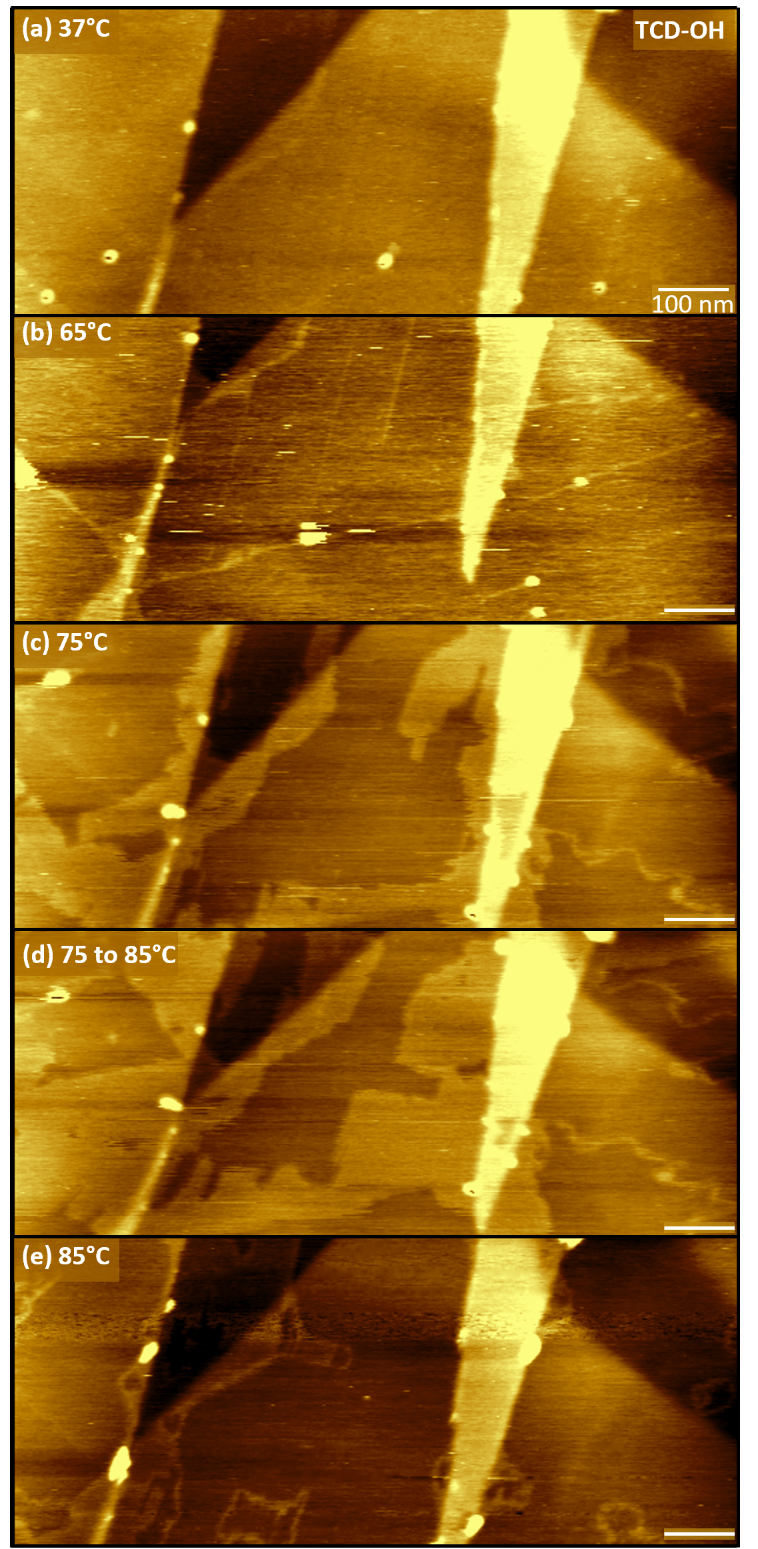


**Figure S17.** Large-scale AFM height images of unpolymerized TCD-OH on HOPG, providing a larger view of areas shown in Figure 6 in the main manuscript. Images depict unpolymerized TCD-OH monolayer on HOPG acquired at 37 °C (a), 65 °C (b), 75 °C (c), 75 to 85 °C (d), and 85 °C (e).


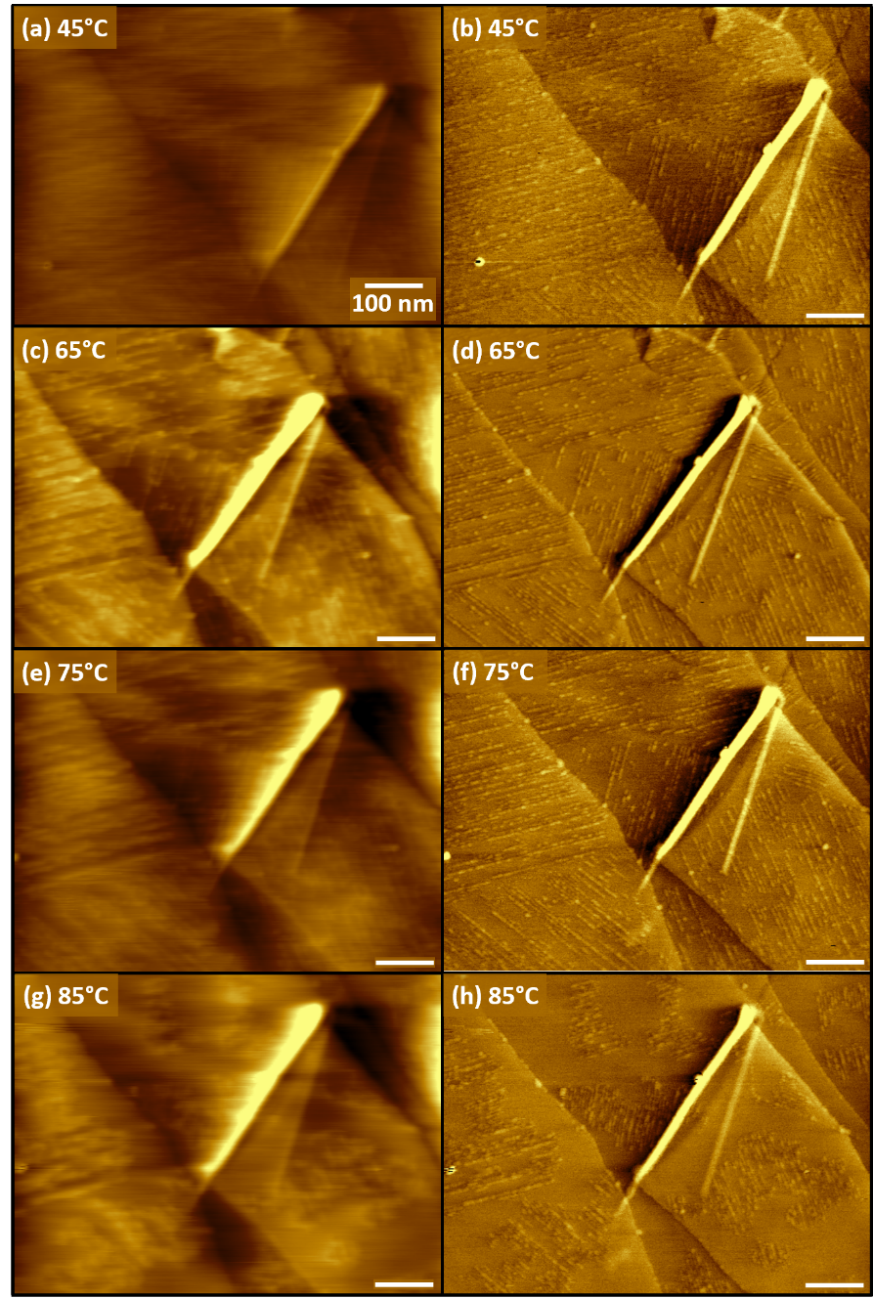


**Figure S18.** AFM height (left column: a, c, e, g) and phase (right column: b, d, f, h) images of a partially polymerized TCD-COOH monolayer prepared at PT65 °C irradiated with UV for 10 min. Sample was imaged at 45 °C (a-b), 65 °C (c-d), 75 °C (e-f), and 85 °C (g-h).


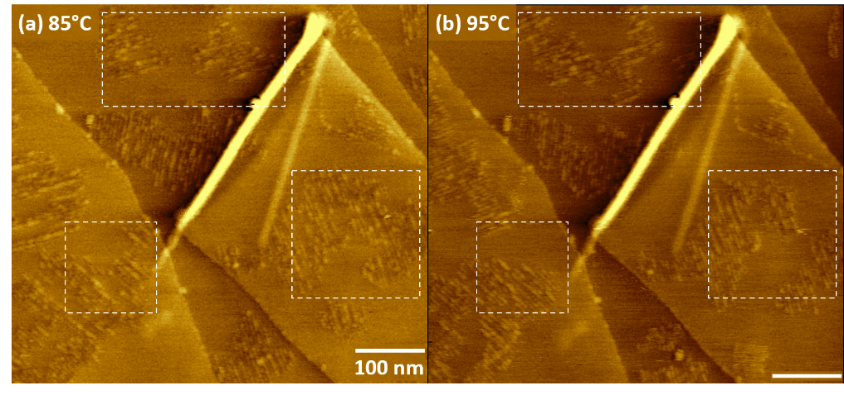


**Figure S19.** AFM phase images of a partially polymerized TCD-COOH monolayer prepared at PT65 °C irradiated with UV for 10 min comparing the monolayer surface at 85 °C and 95 °C. In the white dash boxes, minor shifts in topographical features can be observed.

**
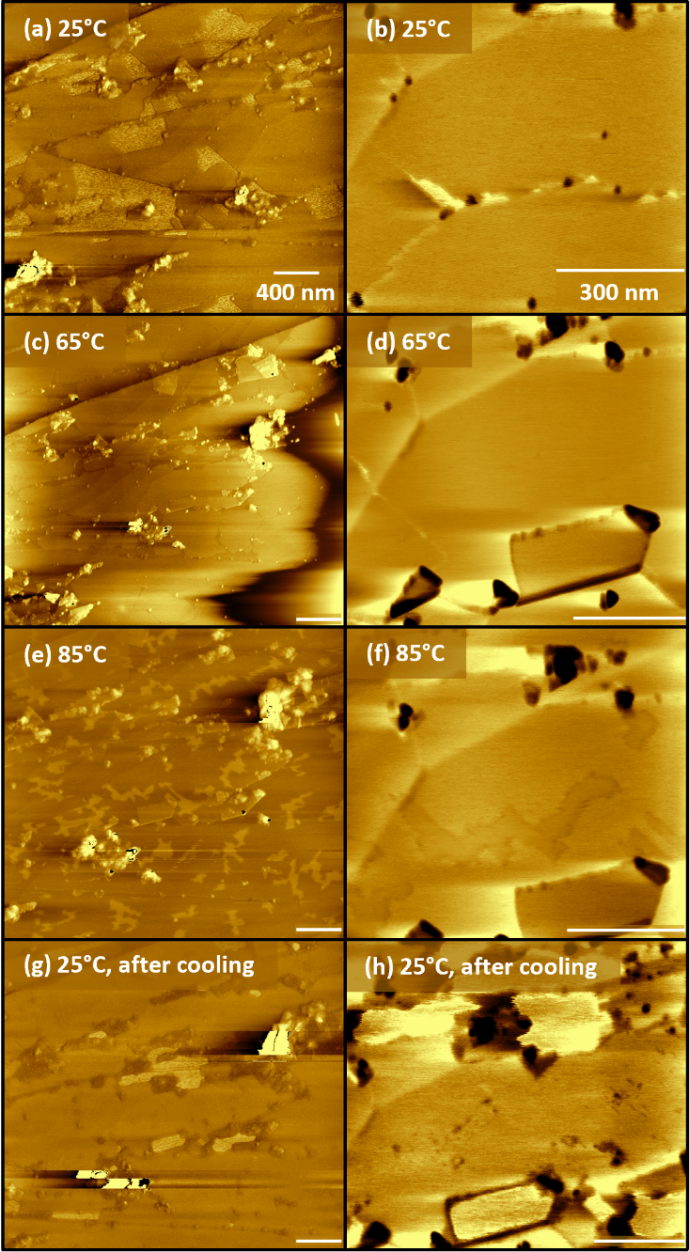
**

**Figure S20.** AFM phase (left column: a, c, e, g) and amplitude (right column: b, d, f, h) images of an unpolymerized TCD-COOH monolayer taken at 25 °C (a-b), 65 °C (c-d), 85 °C (e-f), and 25 °C (g-f) after steady cooling from 85 °C.

**AFM images of partly polymerized TCD-OH, illustrating lifted PDA backbone structure.**

As described in the main manuscript, diacetylene polymerization in striped phase monolayers on HOPG is often characterized based on the formation of 0.15 nm high linear topographical protrusions corresponding to the evolution of a ‘lifted’ PDA backbone. This conformation occurs to avoid steric clashes between relatively long flanking alkyl chain segments in molecules like TCD-COOH and the structural analogues TCD-OH and TCD-NH_2_ utilized in the present work. Since we have shown AFM images of the lifted backbones of TCD-COOH and TCD-NH_2_ in prior work aimed at understanding room temperature polymerization^11-12^, we show the lifted PDA backbone for TCD-OH (Figure S17). The line scane in Figure S19a is acquired at the position of the blue line near the upper center of the image, and illustrates the smaller topographical features corresponding to unpolymerized lamellae (on the left of the line scan) as well as a lifted PDA backbone feature near the right edge of the line scan. Such features become increasingly abundant in images as polymerization time is increased (e.g. 15 min UV exposure in panel (b) compared with 10 min UV exposure in panel (a)).

**
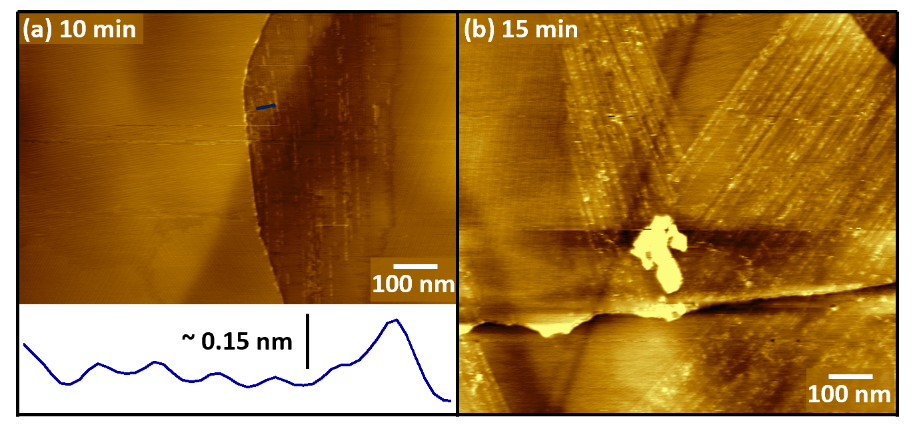
**

**Figure S21.** AFM height images of partially polymerized TCD-OH monolayers irradiated with UV for (a) 10 min and (b) 15 min. Line scan in panel (a) was acquired at the position of the blue line near the upper center of the main image panel, and illustrates the periodic features characteristic of unpolymerized lamellae, as well as a 0.15 nm topographical protrusion near the right of the line scan, where the line intersects a lifted PDA backbone.

**Confocal spectra of fully-polymerized TCD-NH2 and TCD-OH monolayers transferred to PDMS.** Monolayers fully photopolymerized at the indicated temperatures were then transferred to PDMS, and the fluorescence intensities were compared (see main manuscript for discussion. Here we include the full spectra for visual inspection.


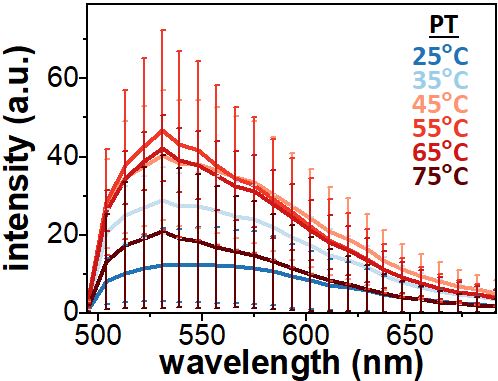


**Figure S22.** Confocal spectra of TCD-NH_2_ for photopolymerization temperature conditions ranging from 25 °C to 75 °C.

**
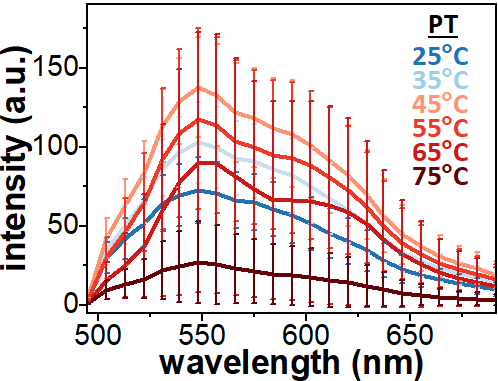
**

**Figure S23.** Confocal spectra of TCD-OH for photopolymerization temperature conditions ranging from 25 °C to 75 °C.

**Comparison with multilayer PDA thermochromism.** To provide context for conventional diacetylene thermochromism measurements, multilayer (optically thick) films of the three monomers were prepared by drop-casting (~20 μL of a 15 mg/mL chloroform solution) onto glass substrates, overnight drying under vacuum, followed by UV irradiation (2 min for TCD-COOH and TCD-NH_2_; 6 min for TCD-OH) and subsequent heating to monitor color transitions, which were photographically documented (Figure S24). Heating was performed using the same Peltier thermoelectric stage employed for the monolayer experiments described above.

Two important distinctions should be noted. First, in the experiments discussed throughout the main text, the *monomer* monolayer is heated prior to polymerization, whereas conventional thermochromism measurements involve heating a *pre-formed polymer* film or crystal. These measurements are therefore not directly analogous, but are included to provide a qualitative comparison. Second, the molecular packing differs substantially: in the monolayer, alkyl chains lie flat in direct contact with the HOPG substrate, whereas in multilayer films the chains are surrounded by neighboring molecules in a three-dimensional environment.

For TCD-COOH, the multilayer polymer film exhibits a thermochromic transition over 55–75 °C, with the most pronounced blue-to-red transition occurring near the upper end of this range. This transition begins near the bulk monomer melting temperature (~56 °C). Notably, the temperature of maximum on-surface (monolayer) polymerization efficiency (65 °C) falls within this thermochromic window, while the onset of visible monolayer disordering on HOPG (~75 °C) coincides with the upper bound of the bulk transition range.

For TCD-NH₂, the thermochromic transition spans 35–55 °C. The bulk melting temperature (~27 °C) lies below this range, and the temperature of maximum surface polymerization efficiency (~45 °C) occurs near its midpoint. In contrast, monolayers on HOPG exhibit progressive disordering over a higher range (45–65 °C), indicating more modest surface stabilization relative to TCD-COOH.

TCD-OH displays comparatively weak bulk chromism under these conditions. After 6 min UV exposure, only faint coloration is observed, with minimal additional development upon extended irradiation (up to 15 min). Blue coloration becomes apparent near 55 °C as the unpolymerized monomer melts (bulk melting temperature ~51 °C), and the multilayer thermochromic transition spans approximately 55–75 °C. In contrast, the temperature of maximum surface polymerization efficiency (~45 °C) lies below this range, while monolayer disordering on HOPG occurs at significantly higher temperatures (75–85 °C). Although detailed structural characterization of the multilayer film is beyond scope, long-chain hydroxyalkanes are known to form both perpendicular (polymerizable) and herringbone (less polymerizable) phases. Preferential stabilization of the perpendicular phase on HOPG may account for the increased surface polymerization observed for TCD-OH relative to multilayer films.

*
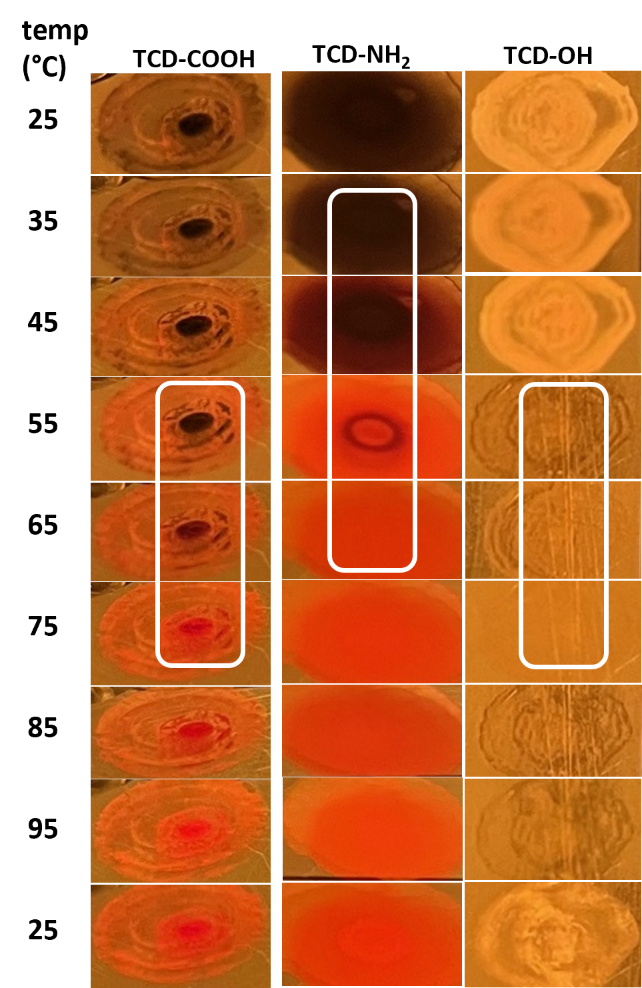
*

**Figure S24.** Digital photographs of 15 mg/mL amphiphile solution drop-cast on a glass slide, exposed to UV irradiation to polymerize (2 min for TCD-COOH and TCD-NH_2_; 6 min for TCD-OH), and heated to the indicated temperature.

**NMR Spectra**

**
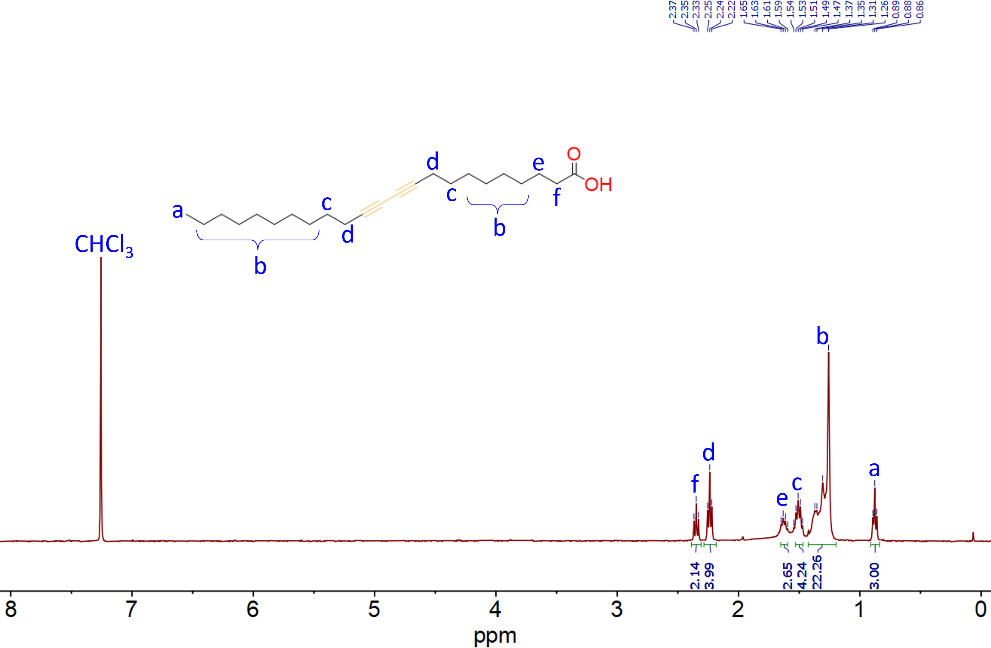
**

**Figure S25.** Full ^1^H NMR spectrum of 10,12-tricosadiynoic acid (TCD-COOH).


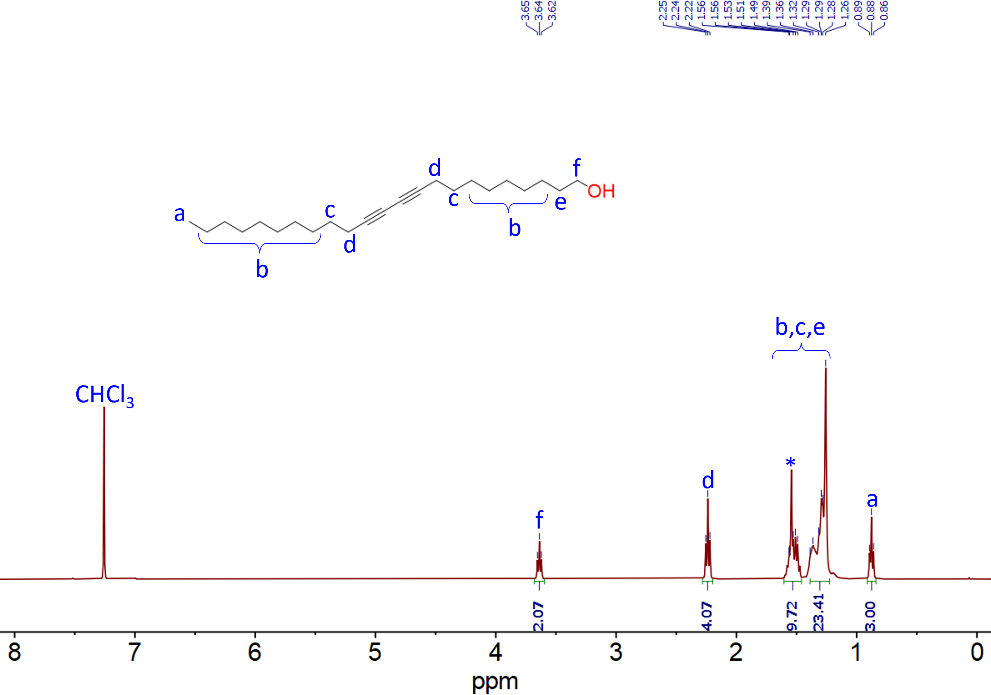


**Figure S26.** Full ^1^H NMR spectrum of 10,12-tricosadiynol (TCD-OH).


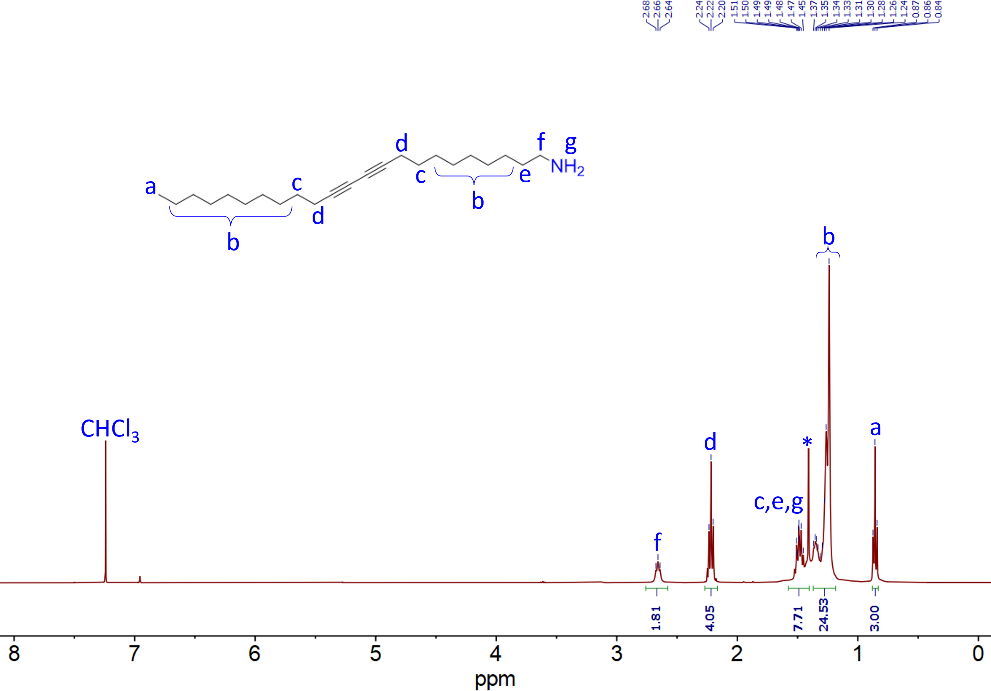


**Figure S27.** Full ^1^H NMR spectrum of 10,12-tricosalkyldiynamine (TCD-NH_2_).

**References cited:**

1. Singh, A.; Arango, J. C.; Shi, A.; d'Aliberti, J. B.; Claridge, S. A. Surface-Templated Glycopolymer Nanopatterns Transferred to Hydrogels for Designed Multivalent Carbohydrate–Lectin Interactions across Length Scales. *J. Am. Chem. Soc.* **2023,** *145*, 1668–1677.

2. Howarth, N. M.; Lindsell, W. E.; Murray, E.; Preston, P. N. Lipophilic Peptide Nucleic Acids Containing a 1,3-Diyne Function: Synthesis, Characterization and Production of Derived Polydiacetylene Liposomes. *Tetrahedron* **2005,** *61*, 8875–8887.

3. Lee, J. P.; Hwang, H.; Chae, S.; Kim, J.-M. A Reversibly Mechanochromic Conjugated Polymer. *Chem. Commun.* **2019,** *55*, 9395–9398.

4. Bang, J. J.; Rupp, K. K.; Russell, S. R.; Choong, S. W.; Claridge, S. A. Sitting Phases of Polymerizable Amphiphiles for Controlled Functionalization of Layered Materials. *J. Am. Chem. Soc* **2016,** *138*, 4448–4457.

5. Davis, T. C.; Bang, J. J.; Brooks, J. T.; McMillan, D. G.; Claridge, S. A. Hierarchical Noncovalent Functionalization of 2D Materials by Controlled Langmuir-Schaefer Conversion. *Langmuir* **2018,** *34*, 1353–1362.

6. Bang, J. J.; Porter, A. G.; Davis, T. C.; Hayes, T. R.; Claridge, S. A. Spatially Controlled Noncovalent Functionalization of 2D Materials Based on Molecular Architecture *Langmuir* **2018,** *34*, 5454–5463.

7. Davis, T. C.; Bechtold, J. O.; Shi, A.; Lang, E. N.; Singh, A.; Claridge, S. A. One Nanometer Wide Functional Patterns with a Sub-10 Nanometer Pitch Transferred to an Amorphous Elastomeric Material. *ACS Nano* **2021,** *15*, 1426–1435.

8. Spagnoli, S.; Briand, E.; Vickridge, I.; Fave, J. L.; Schott, M. Method for Determining the Polymer Content in Nonsoluble Polydiacetylene Films: Application to Pentacosadiynoic Acid. *Langmuir* **2017,** *33*, 1419–1426.

9. Okawa, Y.; Aono, M. Linear Chain Polymerization Initiated by a Scanning Tunneling Microscope Tip at Designated Positions. *J. Chem. Phys.* **2001,** *115*, 2317–2322.

10. Cyr, D. M.; Venkataraman, B.; Flynn, G. W.; Black, A.; Whitesides, G. M. Functional Group Identification in Scanning Tunneling Microscopy of Molecular Adsorbates. *J. Phys. Chem.* **1996,** *100*, 13747–13759.

11. Shi, A.; Villarreal, T. A.; Singh, A.; Hayes, T. R.; Davis, T. C.; Brooks, J. T.; Claridge, S. A. Plenty of Room at the Top: A Multi-Scale Understanding of nm-Resolution Polymer Patterning on 2D Materials. *Angew. Chem., Int. Ed.* **2021,** *60*, 25436–25444.

12. Shi, A.; Singh, A.; Williams, L. O.; Arango, J. C.; Claridge, S. A. Nanometer-Scale Precision Polymer Patterning of PDMS: Multiscale Insights into Patterning Efficiency Using Alkyldiynamines. *ACS Appl. Mater. Interfaces* **2022,** *14*, 22634–22642.
